# Click chemistry oligomerisation of azido-alkyne-functionalised galactose accesses triazole-linked linear oligomers and macrocycles that inhibit *Trypanosoma cruzi* macrophage invasion

**DOI:** 10.1016/j.tet.2015.04.085

**Published:** 2015-09-30

**Authors:** Vanessa L. Campo, Irina M. Ivanova, Ivone Carvalho, Carla D. Lopes, Zumira A. Carneiro, Gerhard Saalbach, Sergio Schenkman, João Santana da Silva, Sergey A. Nepogodiev, Robert A. Field

**Affiliations:** a Faculdade de Ciências Farmacêuticas de Ribeirão Preto, USP, Av. Café S/N, CEP 14040-903, Ribeirão Preto, SP, Brazil; b Department of Biological Chemistry, John Innes Centre, Norwich Research Park, Norwich NR4 7UH, UK; c Faculdade de Medicina de Ribeirão Preto, Department of Parasitology Microbiology and Immunology, USP, Av. Bandeirantes 3900, CEP 14049-900, Ribeirão Preto, SP, Brazil; d Department of Microbiology, Immunology and Parasitology, Universidade Federal de São Paulo, Rua Botucatu 862 8, Andar 04023-062, São Paulo, SP, Brazil

**Keywords:** Click chemistry, Triazole-linked oligomers, *Pseudo*-glycomacrocycles, *Trypanosoma cruzi*, Macrophage invasion

## Abstract

Reaction of 2-(2-(2-azidoethoxy)ethoxy)ethyl 6-*O*-(prop-2-ynyl)-β-d-galactopyranoside (**7**) under CuAAC conditions gives rise to mixed cyclic and linear triazole-linked oligomers, with individual compounds up to d.p. 5 isolable, along with mixed larger oligomers. The linear compounds resolve *en bloc* from the cyclic materials by RP HPLC, but are separable by gel permeation chromatography. The triazole-linked oligomers—*pseudo*-galactooligomers—were demonstrated to be acceptor substrates for the multi-copy cell surface *trans*-sialidase of the human parasite *Trypanosoma cruzi*. In addition, these multivalent TcTS ligands were able to block macrophage invasion by *T. cruzi*.

## Graphical abstract

**Figure:**
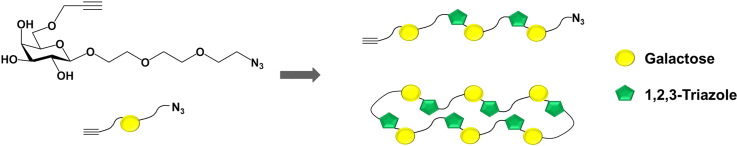

## 1 Introduction

The blood-borne protozoan parasite *Trypanosoma cruzi* causes Chagas' disease, a debilitating and often lethal condition that afflicts millions of people in South and Central America. There is a desperate need for new treatments for this disease, with a requirement for the identification of new drug targets and potential therapeutic agents.^1^, ^2^, ^3^ The biology of carbohydrates—glycobiology^4^—presents many underexplored targets and opportunities^5^, ^6^ for drug discovery.^7^ Including *T. cruzi*^8^ makes extensive use of cell surface mucin glycoproteins^9^ and associated enzyme activities, *trans*-sialidases, in its' attempt to evade the human immune response of one hand, and to adhere to and invade host cells on the other.^10^, ^11^ *T. cruzi trans*-sialidase (TcTS) is considered a relevant drug target for Chagas' disease.^12^, ^13^ With this in mind, we have investigated glycopeptide substrates for and inhibitors of TcTS,^14^, ^15^ including the application of CuAAC click chemistry^16^ with carbohydrate building blocks^17^, ^18^, ^19^ to access libraries of small molecule inhibitors^20^, ^21^, ^22^ and multivalent TcTS ligands displayed on calixarene cores.^23^

**Fig. 1:**
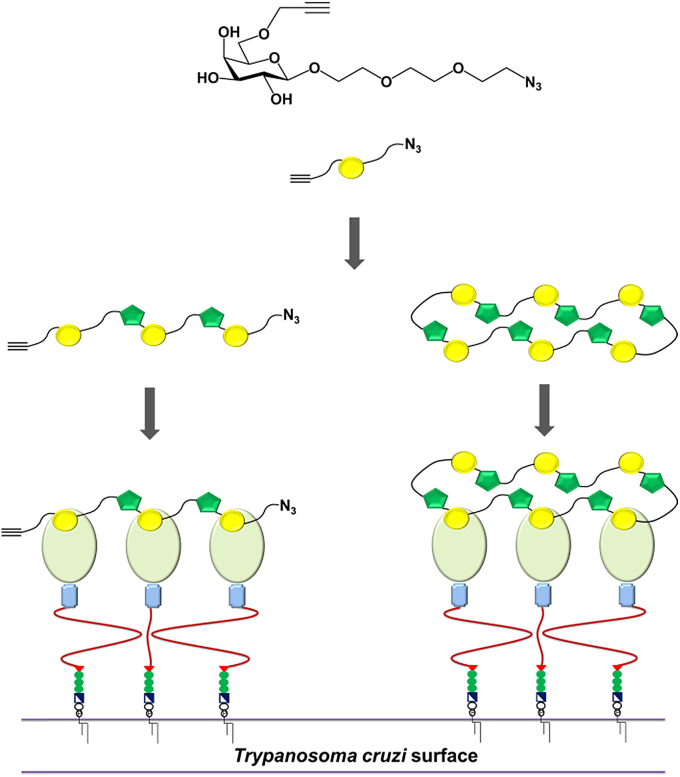
Schematic representation of cell surface-presented *Trypanosoma cruzi trans*-sialidase and potential for its' inhibition by 1,2,3-triazole-linked linear and cyclic *pseudo*-galactooligomers.

There is much current interest in the use of multivalent glycoconjugates as anti-pathogen agents^18^, ^24^, ^25^ and click chemistry presents interesting opportunities for the generation of oligomeric triazole-linked structures.^26^, ^27^, (b) CuAAC click chemistry has found application in the synthesis of analogues of cyclic oligosaccharides,^27^ with medium size macrocycles being prepared in moderate to good yields starting both from protected^28^, ^29^ and ‘free’ sugar derivatives.^30^, ^31^ There are only a few examples of the successful oligomerisation/polymerisation of such azide-alkyne-functionalised carbohydrates leading to linear oligomers, including recent proximity-driven click-polymerization of an 4-*O*-propargyl-β-d-galactopyranosyl azide in a crystal lattice^32^, ^33^ and click-polymerisation of an open chain azido-alkyne derivative of gluconamide.^34^ We have previously shown that the cyclooligomerisation of azido-alkyne-functionalised sugars gives rise to relatively rigid 1,6-linked cyclic *pseudo*-galactooligosaccharides that are recognised by TcTS.^31^ In the present study, we wished to access linear oligomers and/or larger macrocycles, in order to span a greater area that might more adequately map to the distribution of *trans*-sialidase on the parasite cell surface, so blocking *trans*-sialidase-action and associated infection processes^35^ (Fig. 1). Here we have designed linkers between sugar and azide functionality, in contrast to our previous work. We opted for ethylene glycol-based linkers to reduce the potential for non-specific interactions with proteins and membrane components.^36^ We anticipated that these flexible linkers might also promote oligomerisation at the expense of cyclisation. Here we report the chemical synthesis of azido-alkyne-functionalised galactose monomer **7**, CuAAC click reactions thereof and characterisation of the resulting triazole-linked oligomers. Finally, assessment of the interaction of these triazole-linked materials with TcTS is reported, along with initial assessment of their ability to block macrophage invasion by *T. cruzi* parasites.

## 2 Results and discussion

### 2.1 Synthesis of azido-alkyne galactose-containing monomer

**Scheme 1:**
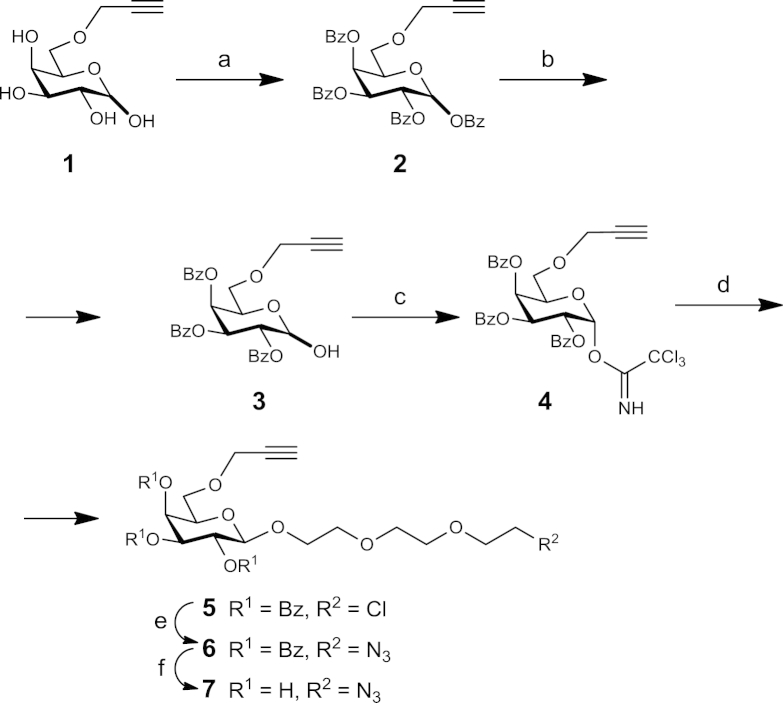
Synthetic route to *azido-alkyne-containing galactose* monomer **7**. Reagents and conditions: a) BzCl, Py, 81%; b) NH_3_, MeOH/THF (7:3), 64%; c) Cl_3_CCN, DBU, CH_2_Cl_2_, 85%; d) 2-(2-(2-chloroethoxy)ethoxy)ethanol, TMSOTf, CH_2_Cl_2_, 85%; e) NaN_3_, NaI, DMF, 97%; f) NaOMe, MeOH, 92%.

The azido-alkyne-substituted galactose monomer **7** was synthesised in nine steps starting from known 6-*O*-propargyl-d-galactopyranose **1**,^31^ as outlined in Scheme 1. Propargyl ether **1** was per-*O*-benzyolated and converted into the required hemiacetal **3** using a solution of ammonia in methanol-THF. The resulting hemiacetal **3** was treated with trichloroacetonitrile and DBU to obtain the corresponding imidate donor **4**, which upon activation with trimethylsilyl trifluoromethanesulfonate (TMSOTf) in the presence of 2-(2-(2-chloroethoxy)ethoxy)ethanol gave chlorinated β-glycoside **5** in a respectable 85% yield for the glycosylation step. In the presence of NaN_3_ and NaI, the chlorinated β-galactoside **5** was converted into the azido β-galactoside **6** in near quantitative yield.

The presence of azide functionality in **6** was evident from a characteristic signal in the IR spectrum [2107 cm^−1^] and the β-configuration followed from the anomeric proton signal (*δ* 5.75, *J*_1,2_=8.0 Hz) in the ^1^H NMR spectrum. De-O-benzoylation of **6** afforded target azido-alkyne-functionalised galactose monomer **7** in an overall yield of 25% from 6-*O*-propargyl galactopyranose **1**.^37^

### 2.2 Cyclisation and oligomerisation through CuAAC reactions based on azido-alkyne-containing galactose monomer 7

**Fig. 2:**
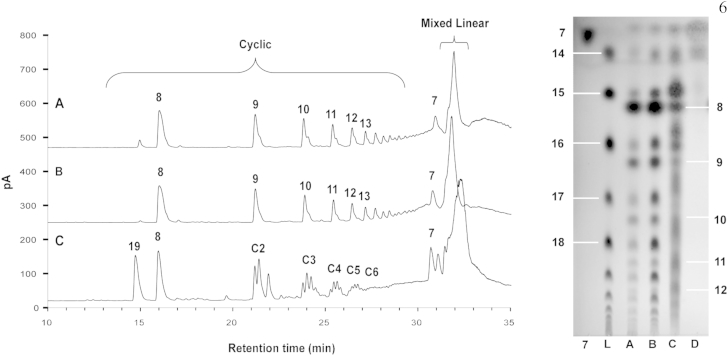
Reverse phase HPLC and normal phase TLC analyses of cyclic and linear products obtained from 1,3 azido-alkyne cycloaddition reactions of monomer **7** (1M in DMF). HPLC trace/TLC lane: **A**, Method A (Cu(I), 110 °C); **B**, Method B (Cu(I), room temperature); **C**, Method C (110 °C); D, Method **D** (room temperature); TLC lane **7**, starting monomer **7**; TLC lane **L**, purified mixed linear oligomer fraction.

**Scheme 2:**
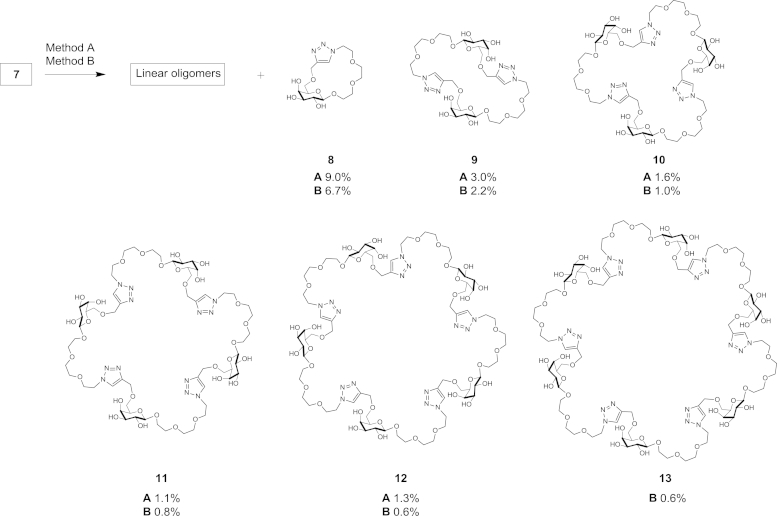
Cyclisation and oligomerisation of monomer **7** (1M in DMF), through CuAAC employing Method A (Cu(I), 110 °C) and Method B (Cu(I), room temperature). Yields (%) for Method A and Method B.

Once synthesised, the reactivity of azido-alkyne-functionalised galactose monomer **7** was tested under CuAAC conditions—at 1 M concentration in DMF with CuSO_4_/Cu turnings either at 110 °C (using microwave irradiation; Method A) or at ambient temperature (Method B). The progress of both reactions could conveniently be followed by TLC analysis. The reaction using Method A was complete after 30 min, compared to 2 days for Method B. TLC analysis showed formation of multiple common products, consistent with the intended oligomerisation, while different relative spot intensities were evident from the two methods (Fig. 2, TLC lanes A and B). The reaction mixtures from both methods were concentrated under reduced pressure, redissolved in water and submitted to reverse phase (RP) HPLC purification. This allowed straightforward separation of cyclic from linear products, as well as resolution of the variously sized cyclic oligomers from each other (Fig. 2, HPLC traces A and B) resulting in samples of cyclic monomer **8**, cyclic dimer **9**, cyclic trimer **10**, cyclic tetramer **11**, cyclic pentamer **12** and cyclic hexamer **13**, along with mixed linear oligomers, which interestingly did not resolve under the reverse phase HPLC conditions employed to separate the corresponding cyclic materials (Scheme 2). The formation of cyclic 1,4-triazole-linked structures was confirmed by virtue of singlet signals of triazole CH protons at *δ* 8.10 ppm^38^ and the absence of propargyl CH signals at *δ* 2.83 in ^1^H NMR spectra.

**Fig. 3:**
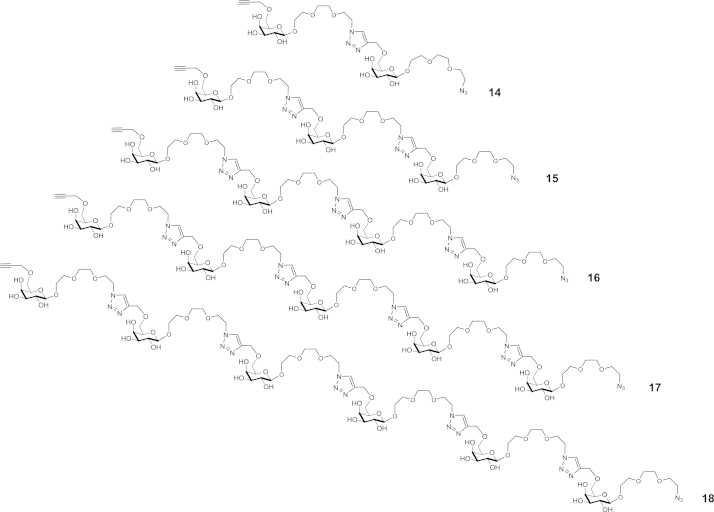
Linear oligomerisation products from the reaction of monomer **7** under CuAAC conditions identified by HRMS compounds **14**–**18** were obtained in a combined yield of 26% (Method A) and 36% (Method B).

The linear oligomeric products eluted on reverse phase HPLC as a single broad peak at ca. 32 min (Fig. 2, HPLC traces A and B). These compounds were well resolved from each other and from the corresponding cyclic oligomers on analytical TLC (Fig. 2, lane L), linear oligomers **14**–**18** have slightly higher R_*f*_ values compared to cyclic product of the same molecular size. Monomer **7** was shown to undergo oligomerisation up to at least a decamer. These analyses alongside isolated yields (see Fig. 2, Fig. 3 and Table S1 in Supplementary data) also illustrate that the lower reaction temperature (room temperature vs 110 °C) favours formation of linear products over the corresponding cyclic isomers. In contrast to reverse phase HPLC, gel permeation chromatography (GPC) on TSK-HW40S enabled separation of linear oligomers up to the pentamer (Fig. 3). It should be noted that these linear compounds contain unreacted azido and alkyne terminal groups capable of further reactions even in the absence of Cu(I) catalyst. This gave rise to complications during handling and storage due to spontaneous cyclisation and oligomerisation of purified compounds (data not shown).

The 1,3-dipolar cycloaddition of azido-alkyne galactose monomer **7** generates series of isomeric cyclic and linear products that have the same molecular formula and hence the same monoisotopic mass.^39^ This was confirmed by high resolution MS analyses of individual isolated cyclic compounds **8**–**13** as well as the mixture of linear oligomers collected as a single peak in HPLC purification (Fig. 2, HPLC traces A and B; TLC lane L). Cyclic and linear products from trimer upwards run in MS analyses as multiply charged species, spectra for, which were de-convoluted to obtain monoisotopic masses (Table 1). Cyclic oligomers had distinctive appearances in ^1^H NMR spectra: for centrosymmetric macrocyles **8**–**13** these were represented by relatively simple spectra of the repeat unit compared to more complex spectra, as be expected for linear oligomers **14**–**18**.

**Table 1:** HRMS data of 1,4-triazole-linked cyclic products and linear oligomers

	Calculated *m*/*z* [M+H]^+^	Cyclic compounds	Found *m*/*z* [M+H]^+^	Linear oligomers	Found^a^*m*/*z* [M+H]^+^
Monomer	376.1751	**8**	376.1701	**7**	376.1708
Dimer	751.3357	**9**	751.3334	**14**	751.3351
Trimer	1126.4998	**10**	1126.4961	**15**	1126.4985
Tetramer	1501.6640	**11**	1501.6511	**16**	1501.6631
Pentamer	1876.8281	**12**	1876.8248	**17**	1876.8273
Hexamer	2251.9923	**13**	2251.9792	**18**	2251.9897

a Data obtained from the analysis of the mixture of linear oligomers obtained as a result of reverse-phase HPLC purification.

### 2.3 Uncatalysed 1,3-dipolar cycloaddition of azido-alkyne-containing galactose monomer 7

**Scheme 3:**
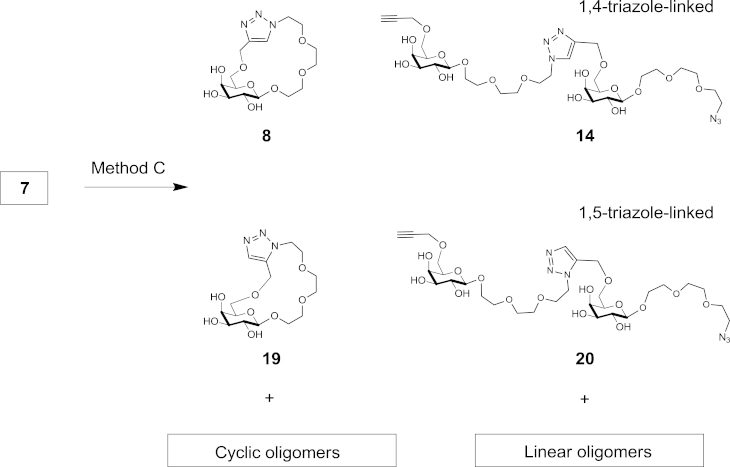
Spontaneous cyclisation and oligomerisation of monomer **7** leading to a mixture of compounds incorporating both 1,4-linked and 1,5-linked 1,2,3-triazole residues.

Given the noted spontaneous but slow 1,3-dipolar cycloaddition of monomer **7** on standing at room temperature (vide supra), the reactivity of **7** at 1 M concentration in DMF at 110 °C (under microwave irradiation; Method C) and at room temperature (Method D) were compared (Scheme 3). Reaction using Method C was complete after 30 min, while Method D gave approximately 10% conversion of monomer **7** after two weeks. TLC analysis showed even more complex multiple product mixtures than the Cu(I)-catalysed reaction (Fig. 2, TLC lanes C and D) due to the expected formation of mixed isomeric 1,4- and 1,5-linked triazoles.

The reaction mixture from Method C was concentrated under reduced pressure, redissolved in water and submitted to the reverse phase HPLC purification. This procedure again allowed separation of cyclic from linear products, as well as resolution of 1,4- and 1,5-linked isomeric cyclic triazole monomer structures **8** and **19** from each other (Fig. 2, HPLC Trace C). Cyclic oligomers eluted on RP HPLC as mixtures of poorly resolved regioisomers, with various combinations of 1,4- and 1,5-linked triazole rings incorporated into macrocycle structures. The ring sizes of mixed 1,4/1,5-linked cyclic dimers (**C2**), mixed 1,4/1,5-linked cyclic trimers (**C3**), mixed 1,4/1,5-linked cyclic tetramers (**C4**), mixed 1,4/1,5-linked cyclic pentamers (**C5**) and mixed 1,4/1,5-linked cyclic hexamer (**C6**) were established by mass spectrometry.

The 1,5-linked cyclic monomer **19** was fully characterised by NMR spectroscopy and HRMS. The formation of the 1,5-linked triazole unit was confirmed by observing the triazole CH singlet signal at *δ* 7.71 and the absence of a propargyl CH signal at *δ* 2.83 in the ^1^H NMR spectra. The 1,4/1,5-linked mixed linear products were submitted to GPC purification on TSK-HW40S in water, which enabled separation of mixed linear products up to a tetramer where linear 1,5-linked triazole dimer **20** and linear 1,4-linked dimer **14**, isolable as single compounds, were characterised by NMR spectroscopy and mass spectrometry.

The linear structures of dimers **14** and **20** were confirmed by NMR spectroscopy, in particular by observation of a methylene signal of the intact propargyl group at *δ* ∼4.18 in the ^1^H NMR spectra. In addition, DTT reduction of azido group in **20** produced amino-terminated compound, which was detected by MS analysis showing an [M+H]^+^ peak at *m*/*z* 725.28, compared to unreduced precursor with an [M+H]^+^ peak at *m*/*z* 751.33. The triazole linkage type in **14** and **20** was also evident from the ^1^H NMR spectra, which showed diagnostic proton resonances of 1,4-linked triazoles at *δ* 8.04 for **14** and of 1,5-linked triazoles at *δ* 7.80 for **20**.^38^

### 2.4 Cyclic triazole-linked oligomers as acceptor substrates for *Trypanosoma cruzi trans*-sialidase (TcTS)

**Scheme 4:**
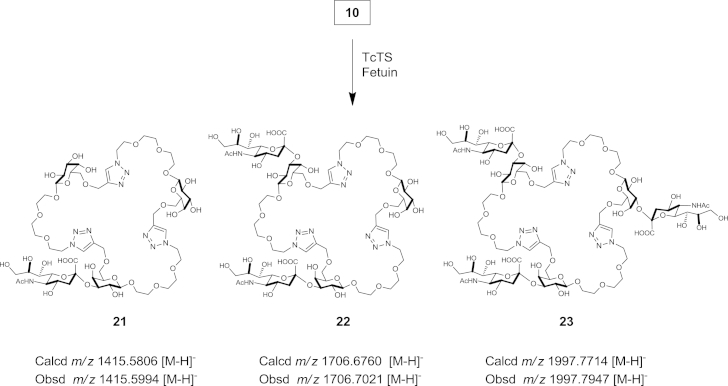
Proposed sialylated products structures **21**, **22** and **23** of enzymatic transformation of compound **10**. Reagents and conditions: 3 mM fetuin, 1 mM compound **10**, *T. cruzi trans*-sialidase in 100 mM, pH 7.5 phosphate buffer, 28 °C, 9 days.

Cyclic compounds **8**–**10** and **19** were tested for their ability to act as substrates for TcTS,^21^, ^40^ with fetuin serving as a donor substrate for *O*-3 sialylation of the galactose residues in the triazole-linked macrocycles. Reactions were carried out over the course of 9 days, with addition of further fetuin and enzyme after 5 days, and addition of fetuin again after 7 days. The reaction mixtures were monitored by TLC and in all cases formation of new compounds was observed, which were identified by HRMS. Thus, reaction of 1,4 triazole-linked cyclic trimer **10** resulted in formation of mono-, di- and tri-sialylated products **21**, **22** and **23**, respectively (Scheme 4). Related reactivity was observed for 1,4-triazole-linked cyclic dimer **9**, which gave two sialylated products, mono-sialylated and di-sialylated derivatives. The 1,4-triazole-linked cyclic monomer **8** and 1,5-triazole-linked cyclic monomer **19** gave mono-sialylated products as expected (see Supplementary data). It is therefore evident that cyclic triazole-linked *pseudo*-galactooligomers are recognised by, and can act as acceptor substrates for, TcTS.

### 2.5 Preliminary biological evaluation of triazole-linked oligomers

As the *trans*-sialidase is an important enzyme associated with host cell invasion by *T. cruzi*,^11^, ^35^ we evaluated the biological potential of our triazole-linked oligomers as blockers of parasite mammalian cell entry. The trypomastigote form of the parasite invades mammalian cells, where it differentiates into amastigote forms that replicate and subsequently de-differentiate to tryposmatigotes. The latter then exit the cell in search of other cells to infect. Our assays were therefore two-fold: assessment of the number of trypomastigotes present in the medium, or assessment of the number of amastigotes inside macrophages.

**Fig. 4:**
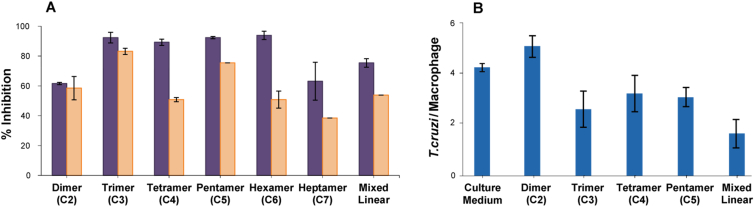
Inhibition of *T. cruzi* invasion of bovine macrophages in the presence of 1,4/1,5-triazole-linked cyclic dimers (C2), trimer (C3), tetramer (C4), pentamer (C5), hexamer (C6), heptamer (C7) and a series of mixed 1,4/1,5-triazole-linked linear oligomers. A: Parasites were applied to macrophages without () or with () pre-incubation with triazoles and following 6 days incubation the numbers of trypomastigotes in the medium was quantified as % reduction in the number of parasite released from macrophages (% inhibition). B: Parasites and triazoles were applied to macrophages and the number of amastigotes present inside the macrophages was quantified as an average number of parasite per macrophage (*T. cruzi*/Macrophage).

Parasites were mixed with bovine macrophages and triazole-linked oligomers, with or without a pre-incubation of the parasite and oligomers; where a pre-incubation was employed, free triazole-linked oligomers were removed by washing before adding the parasites to macrophages. The parasites were left to invade the macrophages and after 6 days, the trypomastigote form found in the medium was removed and counted. As shown in Fig. 4 A (), the incubation of parasite with triazole-linked oligomers substantially inhibited the invasion of macrophage by parasites (>90% for trimer-hexamer). To rule out a direct impact of the triazole-linked compounds on the macrophages, pre-incubation of parasites with triazoles, plus washing to remove excess triazole, was assessed. While the impact on parasite invasion was more modest than when the triazole was present throughout (Fig. 4 A ), this is to be expected given the dramatic reduction in concentration of the inhibitor in these experiments. Gratifyingly, inhibition of macrophage invasion was still pronounced.

**Fig. 5:**
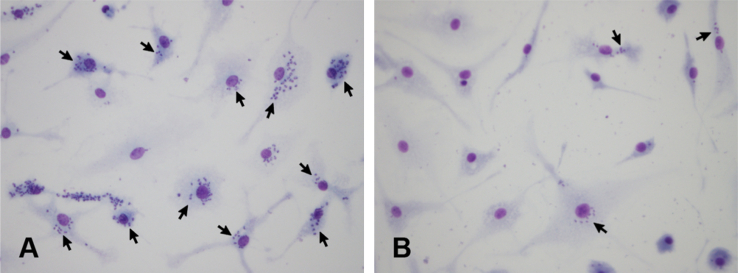
Representative images illustrating amastigote-form parasite numbers present inside macrophages infected with *T. cruzi* in (A) the absence and (B) the presence of 250 μM mixed length 1,4/1,5 triazole-linked linear oligomers. Arrows indicate cells infected with *T. cruzi*.

To complement the above assays, we also assessed differentiated amastigote-form *Trypanosoma cruzi* numbers inside infected macrophages, and the impact of triazole-linked oligomers on these numbers. In keeping with the trypomastigote results (Fig. 4 A), triazole-linked oligomers resulted in a reduction in the number of parasites found inside macrophages (Fig. 4 B; Fig. 5), with a general trend towards larger structures giving greater effect.

Taken together, these data confirm the potential of triazole-linked *trans*-sialidase ligands to block macrophage invasion by *T. cruzi* parasites.

## 3 Conclusions

In this study we have investigated the CuAAC-mediated oligomerisation of an azido-alkyne-functionalised galactose building block **7** for the development of novel oligomeric, triazole-linked compounds with potential for impact against the human parasite *Trypanosoma cruzi*. Despite our initial expectation that the installation of flexible ethylene-glycol-based linker might favour polymerisation of monomer over cyclisation, mixed populations of linear and cyclic triazole-linked oligomers of d.p. 1 to >10 were evident. The cyclic compounds were separable from each other and from the corresponding linear materials by RP HPLC, whilst separation of linear materials up to d.p. 5 could be achieved by gel permeation chromatography. The overall yield of triazole-linked products obtained from polymerisation/cyclooligomerisation of monomer **7** was around 54%, with a higher proportion of linear products (36%) compared to cyclic products (18%) formed in CuAAC reactions conducted at room temperature.

Judicious choice of the sites of galactose modification in monomer **7** (the 1- and 6-positions) results in oligomeric triazole-linked materials that present the 2-, 3- and 4-hydroxyl groups of the sugar units that are required for binding to *T. cruzi trans*-sialidase.^40^ This was borne out by in vitro biotransformations, which established that triazole-linked 1,6-substituted *pseudo*-galactoligomers are indeed recognised as acceptor substrates by *T. cruzi trans*-sialidase. Given the high copy number presentation of *trans*-sialidase on the surface of *T. cruzi*, multivalent ligands for the enzyme might block the surface of the parasite and inhibit TcTS-mediated host cell invasion. Encouragingly, preliminary assessment of linear and cyclic triazole-linked *pseudo*-galactooligomers clearly establishes their ability to block *T. cruzi* invasion of macrophages, with distinct advantage demonstrated by pre-incubation of triazole-linked oligomers with parasite prior to exposure to macrophages. While this phenomenon would be difficult to reproduce medicinally, it suggests that there is scope for optimisation of ligand presentation. The versatile nature of CuAAC-based oligomerisation approach offers significant scope in this regard.

## 4 Experimental section

Chemicals were purchased as reagent grade and used without further purification. Ammonia solution (7N NH_3_ in methanol) and methanolic sodium methoxide (0.5M NaOMe in methanol) were purchased from Sigma Aldrich. All moisture-sensitive reactions were performed under a dry nitrogen atmosphere using oven-dried glassware. Anhydrous solvents were purchased from Sigma Aldrich and dichloromethane was freshly distilled from CaH_2_ prior to use. Microwave-assisted reactions were carried out in Biotage Initiator microwave. Reactions were monitored by thin-layer chromatography (TLC) on aluminium-backed, pre-coated silica gel plates (Silica Gel 60 F_254_, E. Merck) with the indicated eluents. Compounds were visualised under UV light (*λ* 254 nm) and by dipping in ethanol-sulfuric acid (95:5, v/v) followed by heating. Flash chromatography was performed on a Biotage Isolera MPLC system using pre-packed silica gel cartridges. HPLC was carried out using a Dionex system equipped with a Corona Charged Aerosol (CAD) detector. Gel permeation chromatography was performed on a TSK-HW40S column (1.6×80 cm) eluted with water at 0.4 mL/min flow rate. Optical rotations were measured at 20 °C in 1 mL cell in the stated solvent using a Perkin–Elmer 341 polarimeter equipped with a sodium lamp. Nuclear magnetic resonance spectra were recorded on a Bruker Avance III 400 NMR or Bruker Avance 600 spectrometer at 298 K. Chemical shifts (*δ*) are reported in parts per million (ppm) with respect to internal tetramethylsilane (TMS) in CDCl_3_ and residual HOD signal in D_2_O. NMR signal assignments were made with the aid of COSY and HSQC experiments. High resolution ESI MS data were obtained using a Waters Synapt G2 mass spectrometer; data for multiply charged species were deconvoluted using the Mass X3 program. Infra-red spectra were obtained on a Perkin–Elmer FTIR Spectrum BX instrument equipped with MIRacle single reflection horizontal accessory.

### 4.1 2,3,4-Tri-*O*-benzoyl-6-*O*-(prop-2-ynyl)-1-*O*-trichloroacetimidoyl-α-d-galactopyranose (4)

Benzoyl chloride (25 mL, 215 mmol) was added dropwise to a stirred solution of 6-*O*-propargyl galactose (**1**)^31^ (9.7 g, 43 mmol) in pyridine (95 mL) at 0 °C, the mixture was stirred for 1 h at room temperature and ice-cold water (100 mL) was carefully added. The product was extracted with CH_2_Cl_2_ (3×125 mL), the organic extracts were combined and washed with ice-cold 1M HCl (3×100 mL), saturated aqueous NaHCO_3_ solution (4×100 mL), dried (MgSO_4_), filtered and concentrated under reduced pressure. The obtained residue was purified by flash column chromatography (toluene/EtOAc, 7:3) to give compound **2** (21.9 g, 81%, mixture of α/β-anomers) as a colourless solid. Benzoate **2** (6.6 g, 104 mmol) was dissolved in anhydrous THF (70 mL), 7 N NH_3_ in methanol (23 mL) was added, the solution was stirred for 26 h at room temperature and the solvent was removed under reduced pressure. The residue was purified by flash column chromatography (hexane/EtOAc, 7:3) to give hemiacetal **3** (3.5 g, 64%). Compound **3** (2.8 g, 5.2 mmol) and trichloroacetonitrile (1.9 g, 13.0 mmol) were dissolved in anhydrous CH_2_Cl_2_ (10 mL), the solution was cooled to 0 °C, stirred for 2 h and concentrated under reduced pressure. The residue was purified by flash column chromatography (hexane/EtOAc, 7:3) to afford trichloroacetimidate **4** (2.9 g, 99%) as a colourless syrup. R_*f*_ 0.55 (hexane/EtOAc, 7:3); [α]_D_ +1.2 (*c* 0.6, CHCl_3_); *δ*_H_ (400 MHz, CDCl_3_) 8.61 (1H, s, NH), 8.09–8.07 (2H, m, Ph), 7.96–7.93 (2H, m, Ph), 7.81–7.79 (2H, m, Ph), 7.63 (1H, m, Ph), 7.52–7.24 (8H, m, Ph), 6.88 (1H, d, *J*_1,2_=3.6 Hz, H-1), 6.08 (1H, dd, *J*_3,4_=3.4 Hz, *J*_4,5_=1.3 Hz, H-4), 6.04 (1H, dd, *J*_3,4_=3.4 Hz, *J*_2,3_=10.6 Hz, H-3), 5.92 (1H, dd, *J*_1,2_=3.6 Hz, *J*_2,3_=10.6 Hz, H-2), 4.68 (1H, m, H-5), 4.12 (2H, m, C*H*_*2*_C≡CH), 3.75 (2H, d, *J*=6.2 Hz, H-6a, H-6b), 2.24 (1H, t, *J*=2.3 Hz, CH_2_C≡C*H*); *δ*_C_ (100.6 MHz, CDCl_3_) 165.7–165.4, 133.6–133.2, 129.9–128.3, 93.9, 75.3, 70.3, 68.6, 68.1, 67.5, 67.1, 58.7; HRMS (ESI) *m*/*z* calcd for C_32_H_26_Cl_3_NO_9_Na^+^ ([M+Na]^+^): 696.0571, found: 696.0565.

### 4.2 2-(2-(2-Chloroethoxy)ethoxy)ethyl 6-*O*-(prop-2-ynyl)-2,3,4-tri-*O*-benzoyl-β-d-galactopyranoside (5)

To a solution of imidate **4** (2.9 g, 5.2 mmol) and 2-(2-(2-chloroethoxy)ethoxy)ethanol (730 mg, 4.3 mmol) in anhydrous CH_2_Cl_2_ (50 mL) containing 4 Å mol. sieves TMSOTf (90 μL, 0.5 mmol) was gradually added at −30 °C, the reaction mixture was allowed to warm to room temperature and stirred for 3 h. The reaction was quenched by addition of Et_3_N (1.0 mL) and concentrated under reduced pressure. The residue was purified by flash chromatography (EtOAc/hexane, 7:3) to give compound **5** (2.7 g, 85%) as a colourless solid. R_*f*_ 0.17 (EtOAc/hexane, 7:3); [α]_D_ +0.9 (*c* 0.7, CHCl_3_); IR (neat) 2877, 2360, 1725, 1601, 1584, 1451, 1260, 1094, 750 cm^−1^; *δ*_H_ (400 MHz, CDCl_3_) 8.09–8.07 (2H, m, Ph), 7.98–7.96 (2H, m, Ph), 7.79–7.77 (2H, m, Ph), 7.61 (1H, m, Ph), 7.53–7.36 (8H, m, Ph), 5.90 (1H, dd, *J*_3,4_=3.5 Hz, *J*_4,5_=1.0 Hz, H-4), 5.75 (1H, dd, *J*_1,2_=8.0 Hz; *J*_2,3_=10.5 Hz, H-2), 5.56 (1H, dd, *J*_3,4_=3.5 Hz; *J*_2,3_=10.5 Hz, H-3), 4.90 (1H, d, *J*_1,2_=8.0 Hz, H-1), 4.19–4.12 (3H, m, H-5, C*H*_*2*_C≡CH), 4.11–4.04 (1H, m, GalOC*H*_*2*_), 3.87–3.81 (1H, m, GalOC*H*_*2*_), 3.76 (2H, m, H-6a, H-6b), 3.69–3.60 (4H, m, C*H*_*2*_), 3.59–3.53 (2H, m, CH_2_Cl_2_), 3.51–3.47 (2H, m, C*H*_*2*_), 3.44–3.38 (2H, m, C*H*_*2*_), 2.30 (1H, t, *J*=2.3 Hz, CH_2_C≡C*H*); *δ*_C_ (100.6 MHz, CDCl_3_) *δ* 165.6–165.2, 133.4–133.2, 130.0–128.3, 101.7, 79.1, 75.0, 72.7, 71.9, 71.3, 70.7, 70.5, 70.4, 69.9, 69.5, 68.5, 67.8, 58.7, 42.7; HRMS (EI) *m*/*z* calcd for C_36_H_37_ClO_11_Na^+^ ([M+Na]^+^): 703.1922, found: 703.1917.

### 4.3 2-(2-(2-Azidoethoxy)ethoxy)ethyl 6-*O*-(prop-2-ynyl)-2,3,4-tri-*O*-benzoyl-β-d-galactopyranoside (6)

To a solution of β-galactoside **5** (5.2 g, 7.6 mmol) in DMF (50 mL) NaN_3_ (2.5 g, 38.0 mmol) and NaI (1.1 g, 8.0 mmol) were added and the reaction mixture was stirred at 70 °C for 28 h. The reaction mixture was diluted with water (20 mL) and extracted with CH_2_Cl_2_ (3×30 mL). The resulting organic extracts were dried (MgSO_4_), and concentrated under reduced pressure to give compound **6** (5.0 g, 97%) as a colourless syrup. R_*f*_ 0.17 (hexane/EtOAc, 7:3); [α]_D_ +0.9 (*c* 0.7, CHCl_3_); IR (neat) 2874, 2359, 2107, 1724, 1601, 1584, 1451, 1259, 1094, 752 cm^−1^; *δ*_H_ (400 MHz, CDCl_3_) 8.09–8.07 (2H, m, Ph), 7.98–7.96 (2H, m, Ph), 7.79–7.77 (2H, m, Ph), 7.61 (1H, m, Ph), 7.53–7.36 (8H, m, Ph), 5.90 (1H, dd, *J*_3,4_=3.5 Hz, *J*_4,5_=1.0 Hz, H-4), 5.75 (1H, dd, *J*_1,2_=8.0 Hz; *J*_2,3_=10.5 Hz, H-2), 5.56 (1H, dd, *J*_3,4_=3.5 Hz; *J*_2,3_=10.5 Hz, H-3), 4.90 (1H, d, *J*_1,2_=8.0 Hz, H-1), 4.19–4.12 (3H, m, H-5, C*H*_*2*_C≡CH), 4.11–4.04 (1H, m, GalOC*H*_*2*_), 3.87–3.81 (1H, m, GalOC*H*_*2*_), 3.76 (2H, m, H-6a, H-6b), 3.69–3.59 (2H, m, C*H*_*2*_), 3.57–3.52 (2H, m, C*H*_*2*_), 3.52–3.46 (2H, m, C*H*_*2*_), 3.44–3.37 (2H, m, C*H*_*2*_), 3.51–3.29 (2H, m, C*H*_*2*_N_3_), 2.30 (1H, t, *J*=2.3 Hz, CH_2_C≡C*H*); *δ*_C_ (100.6 MHz, CDCl_3_) 165.6–165.2, 133.4–133.2, 130.0–128.3, 101.5, 79.1, 75.0, 72.7, 71.9, 71.3, 70.7, 70.5, 70.4, 69.95, 69.9, 69.5, 68.5, 67.8, 58.7, 50.6; HRMS (ESI) *m*/*z* calcd for C_36_H_37_N_3_O_11_Na^+^ ([M+Na]^+^): 710.2326, found: 710.2320.

### 4.4 2-(2-(2-Azidoethoxy)ethoxy)ethyl 6-*O*-(prop-2-ynyl)-β-d-galactopyranoside (7)

A solution of compound **6** (2.4 g, 3.48 mmol) in absolute MeOH (20 mL) was treated with 0.5 M NaOMe in MeOH (2.7 mL, 1.39 mmol), the solution was kept at room temperature for 1 h, neutralized with dry ice, concentrated under reduced pressure and purified by flash chromatography (CH_2_Cl_2_/MeOH, 9:1) to give compound **7** (1.2 g, 91%) as a colourless syrup. [α]_D_ −1.6 (*c* 1.3, MeOH); *δ*_H_ (600 MHz, Methanol-*d*_4_) 4.16 (1H, d, *J*_1,2_=7.6 Hz, H-1), 4.11 (2H, d, *J*=2.4 Hz, C*H*_2_C≡CH), 3.94–3.84 (1H, m, H-6a), 3.69 (1H, dd, *J*_3,4_=3.4 Hz, *J*_4,5_=1.1 Hz, H-4), 3.67–3.54 (12H, m, H-5, H-6b, CH_2_), 3.43 (1H, dd, *J*_1,2_=7.6 Hz, *J*_2,3_=9.7 Hz, H-2), 3.37 (1H, dd, *J*_2,3_=9.7 Hz, *J*_3,4_=3.4 Hz, H-3), 3.28 (2H, t, *J*=5.0 Hz, C*H*_*2*_N_3_), 2.76 (1H, t, *J*=2.4 Hz, CH_2_C≡C*H*); *δ*_C_ (100.6 MHz, D_2_O) 105.0, 80.6, 76.0, 75.0, 74.7, 72.5, 71.6 (2C), 71.4, 71.1, 70.4, 70.3, 69.7, 59.3, 51.8; HRMS (ESI) *m*/*z* calcd for C_15_H_25_N_3_O_8_Na^+^ ([M+Na]^+^): 398.1539, found: 398.1534.

### 4.5 Cu(I)-catalysed oligomerisation of monomer 7

A solution of azido-alkyne monomer **7** (100 mg, 0.3 mmol) containing Cu turnings (260 mg, 4.1 mmol) and CuSO_4_ (11 mg, 0.1 mmol) in DMF (0.27 mL) was placed into a sealed microwave tube. The tube was either submitted to microwave irradiation at 110 °C at 50 W magnetron power (*Method A*) or stirred at room temperature for 2 days (*Method B*). The reaction progress was followed by the TLC (CH_3_CN/EtOAc/*i*PrOH/H_2_O, 85:20:50:50) taking samples at 15 min intervals (*Method A*) or 24 h intervals (*Method B*). After completion the reaction mixtures were separated from Cu turnings and the solvent was removed by repeated evaporation with toluene under reduced pressure. The residues were *re*-dissolved in water and subjected to purification using HPLC on Phenomenex Luna C-18 column (10×250 mm). HPLC method included linear gradient from 0% to 35% in 50 min of MeCN in 0.1% aqueous CF_3_CO_2_H at 1 mL/min flow rate. Peaks were detected by a CAD and collected as individual compounds or mixtures of oligomers, which were identified using ^1^H NMR and/or HRMS.

#### 4.5.1 Cyclic monomer **8**

HPLC retention time 16.3 min; yield 9.0 mg (9.0%) (*Method A*), 6.7 mg (6.7%) (*Method B*); *δ*_H_ (400 MHz, D_2_O) 8.15 (1H, s, C*H* triazole), 4.93 (1H, d, *J*=13.4 Hz, C*H*_2_a-triazole), 4.71–4.61 (3H, m, CH_2_b-triazole, CH_2_N), 4.38 (1H, d, *J*_1,2_=7.9 Hz, H-1), 4.02–3.94 (3H, m, OC*H*_2_CH_2_N, H-6a), 3.91 (1H, d, *J*_3,4_=3.5 Hz, H-4), 3.90–3.66 (10H, m, CH_2_, H-5, H-6b), 3.64 (1H, dd, *J*_2,3_=9.7 Hz; *J*_3,4_=3.5 Hz, H-3), 3.50 (1H, dd, *J*_1,2_=7.9 Hz; *J*_2,3_=9.7 Hz, H-2); *δ*_C_ (100.6 MHz, D_2_O) 143.6, 126.2, 102.2, 74.2, 72.7, 70.6, 69.6, 69.4, 69.4, 69.1, 68.9, 68.5, 62.4, 49.7; HRMS (EI) *m*/*z* calcd for C_15_H_26_N_3_O_8_^+^ [M+H]^+^: 376.1714, found 376.1710.

#### 4.5.2 Cyclic dimer **9**

HPLC retention time 21.3 min; yield 3.0 mg (3.0%) (*Method A*), 2.2 mg (2.2%) (*Method B*); *δ*_H_ (400 MHz, D_2_O) 8.12 (2H, s, C*H* triazole), 4.74 (4H, m, C*H*_2_-triazole), 4.65 (4H, m, CH_2_N), 4.38 (2H, d, *J*_1,2_=7.9 Hz, H-1), 4.02–3.92 (6H, m, OC*H*_2_CH_2_N, H-6a), 3.91 (2H, d, *J*_3,4_=3.6 Hz, H-4), 3.86–3.70 (8H, m, CH_2_, H-5, H-6b), 3.69–3.57 (14H, m, CH_2_, H-3), 3.53 (2H, dd, *J*_1,2_=7.9 Hz, *J*_2,3_=9.9 Hz, H-2); *δ*_C_ (100.6 MHz, D_2_O) 143.7, 125.6, 102.8, 73.5, 72.6, 70.6, 69.6 (2C), 69.4, 68.9, 68.7, 68.6, 63.4, 50.1; HRMS (ESI) *m*/*z* calcd for C_30_H_51_N_6_O_16_ ([M+H]^+^): 751.3357, found: 751.3334.

#### 4.5.3 Cyclic trimer **10**

HPLC retention time 23.9 min; yield 1.6 mg (1.6%) (*Method A*), 1.0 mg (1.0%) (*Method B*); *δ*_H_ (400 MHz, D_2_O) 8.10 (3H, s, C*H* triazole), 4.72 (6H, m, C*H*_2_-triazole), 4.63 (6H, m, CH_2_N), 4.39 (3H, d, *J*_1,2_=7.8 Hz, H-1), 4.00–3.93 (9H, m, OC*H*_2_CH_2_N, H-6a), 3.90 (3H, d, *J*_3,4_=3.4 Hz, H-4), 3.85–3.73 (12H, m, CH_2_, H-5, H-6b), 3.70–3.58 (21H, m, CH_2_, H-3), 3.53 (3H, dd, *J*_1,2_=7.9 Hz, *J*_2,3_=9.9 Hz, H-2); *δ*_C_ (100.6 MHz, D_2_O) 143.8, 125.5, 102.8, 73.5, 72.6, 70.6, 69.7, 69.6 (2C), 69.4, 68.9, 68.7, 63.4, 50.0; HRMS (ESI) *m*/*z* calcd for C_45_H_76_N_9_O_24_ ([M+H]^+^): 1126.4998, found: 1126.4961.

#### 4.5.4 Cyclic tetramer **11**

HPLC retention time 25.5 min; yield 1.1 mg (1.1%) (*Method A*), 0.8 mg (0.8%) (*Method B*); *δ*_H_ (400 MHz, D_2_O) 8.10 (4H, s, C*H* triazole), 4.72 (8H, m, C*H*_2_-triazole), 4.63 (8H, m, CH_2_N), 4.39 (4H, d, *J*_1,2_=7.8 Hz, H-1), 4.00–3.93 (12H, m, OC*H*_2_CH_2_N, H-6a), 3.90 (4H, d, *J*_3,4_=3.4 Hz, H-4), 3.85–3.73 (16H, m, CH_2_, H-5, H-6b), 3.70–3.58 (28H, m, CH_2_, H-3), 3.53 (4H, dd, *J*_1,2_=7.9 Hz, *J*_2,3_=9.9 Hz, H-2); *δ*_C_ (100.6 MHz, D_2_O) 143.8, 125.5, 102.8, 73.5, 72.6, 70.6, 69.7, 69.6, 69.5, 69.4, 68.9, 68.7, 63.4, 50.0; HRMS (ESI) *m*/*z* calcd for C_60_H_101_N_12_O_32_ ([M+H]^+^): 1501.664, found: 1501.6511.

#### 4.5.5 Cyclic pentamer **12**

HPLC retention time 26.5 min; yield 1.3 mg (1.3%) (*Method A*), 0.6 mg (0.6%) (*Method B*); *δ*_H_ (400 MHz, D_2_O) 8.10 (5H, s, C*H* triazole), 4.71 (10H, m, C*H*_2_-triazole), 4.63 (10H, m, CH_2_N), 4.39 (5H, d, *J*_1,2_=7.9 Hz, H-1), 4.01–3.94 (15H, m, OC*H*_2_CH_2_N, H-6a), 3.90 (5H, d, *J*_3,4_=3.4 Hz, H-4), 3.85–3.73 (20H, m, CH_2_, H-5, H-6b), 3.70–3.58 (35H, m, CH_2_, H-3), 3.53 (5H, dd, *J*_1,2_=7.9 Hz, *J*_2,3_=9.9 Hz, H-2); *δ*_C_ (100.6 MHz, D_2_O) 143.8, 125.5, 102.8, 73.5, 72.6, 70.6, 69.6, 69.6, 69.5, 69.4, 68.9, 68.7 (2C), 63.4, 50.0; HRMS (ESI) *m*/*z* calcd for C_75_H_126_N_15_O_40_ ([M+H]^+^): 1876.8281, found: 1876.8248.

#### 4.5.6 Cyclic hexamer **13**

HPLC retention time 27.2 min; yield 0.5 mg (0.5%) (*Method B*); *δ*_H_ (400 MHz, D_2_O) 8.11 (6H, s, C*H* triazole), 4.72 (12H, m, C*H*_2_-triazole), 4.67–4.62 (12H, m, CH_2_N), 4.39 (6H, d, *J*_1,2_=7.8 Hz, H-1), 4.01–3.96 (18H, m, OC*H*_2_CH_2_N, H-6a), 3.90 (6H, d, *J*_3,4_=2.8 Hz, H-4), 3.87–3.71 (24H, m, CH_2_, H-5, H-6b), 3.70–3.59 (42H, m, CH_2_, H-3), 3.53 (6H, dd, *J*_1,2_=7.9 Hz, *J*_2,3_=9.8 Hz, H-2); *δ*_C_ (100.6 MHz, D_2_O) 102.8, 73.4, 72.5, 70.6, 69.5, 69.3, 68.8, 68.7, 63.4, 50.0; HRMS (ESI) *m*/*z* calcd for C_90_H_151_N_18_O_48_ ([M+H]^+^): 2251.9923, found: 2251.9792.

#### 4.5.7 Cyclic oligomers (DP>6)

HPLC retention time 28–30 min; yield 13.7 mg (13.7%) (*Method A*), 6.0 mg (6.0%) (*Method B*).

#### 4.5.8 Linear oligomers

HPLC retention time 32.0 min; yield 25.8 mg (25.8%) (*Method A*), 35.7 mg (35.7%) (*Method B*); HRMS (ESI) *m*/*z* calcd for ([M+H]^+^): 376.1751; 751.3357; 1126.4998; 1501.6640; 1876.8281; 2251.9923, found: 376.1708; 751.3351; 1126.4985; 1501.6631; 1876.8273; 2251.9897.

### 4.6 Uncatalysed oligomerisation of monomer 7

A solution of compound **7** (100 mg, 0.3 mmol) was placed into a sealed microwave tube. The reaction mixture was submitted to microwave irradiation at 110 °C at 50 W magnetron power (*Method C*). The reaction progress was followed by TLC (CH_3_CN/EtOAc/*i*PrOH/H_2_O, 85:20:50:50) taking samples at 15 min intervals (*Method C*). After 30 min the solvent was removed by repeated evaporation with toluene under reduced pressure. The residues were redissolved in water and subjected to purification using HPLC on Phenomenex Luna C-18 column (10×250 mm). HPLC method included linear gradient from 0% to 35% in 50 min of MeCN in 0.1% aqueous CF_3_CO_2_H. Peaks were detected by a CAD detector and collected as individual compounds or mixtures of oligomers, which were identified using ^1^H NMR and/or HRMS (ESI).

#### 4.6.1 Linear dimer **14**

HPLC retention time 32.0 min; yield 1.2 mg (1.2%); *δ*_H_ (400 MHz, D_2_O) 8.02 (s, 1H), 4.60–4.52 (2H, m, C*H*_2_-triazole), 4.36–4.27 (2H, m, H-1, H-1′), 4.23–4.14 (2H, m, C*H*_2_C≡CH), 3.98–3.87 (4H, m, OC*H*_2_CH_2_N), 3.84–3.81 (2H, m, H-4), 3.78–3.52 (26H, m, CH_2_, H-6, H′, H5, H-3), 3.47–3.41 (4H, m, H-2, CH_2_N_3_); *δ*_C_ (100.6 MHz, D_2_O) 102.7, 76.9, 73.4, 72.5, 70.5, 68.7, 68.5, 63.3, 58.1, 50.0, 49.9.

#### 4.6.2 Cyclic monomer **19**

HPLC retention time 14.9 min; yield 8.7 mg (8.7%); *δ*_H_ (400 MHz, D_2_O) 7.88 (1H, s, C*H* triazole), 4.92–4.83 (2H, m, CH_2_-triazole), 4.72–4.56 (2H, m, CH_2_N), 4.42 (1H, d, *J*_1,2_=7.9 Hz, H-1), 4.06–4.02 (2H, m, OC*H*_2_CH_2_N), 3.97–3.82 (6H, m, CH_2_, H-4, H-5,H-6), 3.74–3.57 (7H, m, CH_2_, H-3), 3.53 (1H, dd, *J*_1,2_=7.9 Hz; *J*_2,3_=9.9 Hz, H-2); *δ*_C_ (100.6 MHz, D_2_O) 140.5, 102.2, 74.4, 72.7, 71.1, 70.6, 70.3, 69.7, 69.5, 69.3, 68.8, 61.2, 48.5; HRMS (ESI) *m*/*z* Calcd for C_15_H_26_N_3_O_8_ ([M+H]^+^): 376.1751, found: 376.1701.

#### 4.6.3 Linear dimer **20**

HPLC retention time 32.0 min; yield 1.5 mg (1.5%); *δ*_H_ (400 MHz, D_2_O) 7.77 (1H, s, C*H* triazole), 4.60–4.54 (2H, m, CH_2_N), 4.35–4.29 (2H, m, H-1, H-1′), 4.23–4.14 (2H, m, C*H*_2_C≡CH), 3.97–3.88 (4H, m, OC*H*_2_CH_2_N), 3.84–3.81 (2H, m, H-4),3.78–3.52 (26H, m, CH_2_, H-6, H-5, H-3), 3.47–3.41 (4H, m, H-2, CH_2_N_3_); *δ*_C_ (100.6 MHz, D_2_O) 102.7, 73.1, 72.4, 70.6, 68.7, 68.6, 60.3, 58.1, 50.0, 48.1.

### 4.7 *trans*-Sialidase-mediation sialylation of triazole-linked *pseudo*-galactooligomers

To a solution of fetuin^41^ as a donor substrate (25 μL, 3 mM in 0.1 mM phosphate buffer pH 7.0) and triazole-linked acceptor substrates **8**–**10** and **19** (25 μL, 1 mM in 0.1 mM phosphate buffer pH 7.0) was added TcTS enzyme (25 μL). The mixture was incubated at 28 °C for 5 days when additional 25 μL aliquots of TcTS enzyme and fetuin were added; after a further 2 days additional 25 μL aliquot of fetuin was added. Reaction progress was followed by TLC (CH_3_CN/EtOAc/*i*PrOH/H_2_O, 85:20:50:50). The protein was precipitated by heating the mixture at 100 °C for 1 min and the denatured enzymes were removed by centrifugation (14,000 rpm, 5 min). The supernatant was transferred to a new Eppendorf tube, concentrated by freeze drying and submitted to HRMS analysis.

### 4.8 Macrophage invasion assays

Compounds used in preliminary macrophage invasion assays included a series of mixed 1,4/1,5- triazole-linked cyclic compounds **C2**, **C3**, **C4**, **C5**, **C6**, and **C7** and a mixture of linear 1,4/1,5-triazole-linked oligomers generated by Method C (Section 4.6). *Trypanosoma cruzi* Y strain was cultivated in the LLC-MK2 host cells, as described elsewhere.^42^

#### 4.8.1 Preparation of macrophages

Macrophages derived from bone marrow (BMMOs) were obtained as described previously.^43^ Briefly, total bone marrow cells were cultured in RPMI 1640 medium (Sigma–Aldrich), supplemented with 10% Fetal Bovine Serum (Cutilab) and 30% L-929 cell-conditioned media at 37 °C under 5% CO_2_ atmosphere. On the seventh day of culture BMMOs were harvested and plated in 96 or 24 well microplates (at 5×10^5^ cells/mL).

#### 4.8.2 Preparation of trypomastigotes

Trypomastigote form *Trypanosoma cruzi* Y strain (1.5×10^6^ cells/mL) was added into wells containing BMMOs in two different ways: (Treatment 1) trypomastigotes and triazole-linked compounds at a final concentration of 250 μM were plated at the same time; and (Treatment 2) trypomastigotes and triazole-linked compounds at a final concentration of 250 μM were first pre-incubated for 1 h, then the parasites were washed to remove free compound and were plated to the wells containing BMMOs. In both methods the trypomastigotes were maintained for 2 h in contact with BMMOs. The parasites that had not invaded macrophages were removed by serial washing after the 2 h incubation.

#### 4.8.3 Analysis of released trypomastigotes

After six days incubation, free trypomastigotes that had escaped from macrophages were counted in a Neubauer chamber and results are expressed as a percentage inhibition with respect to trypomastigotes released from infected cells that had not been treated with triazole-linked compounds.

#### 4.8.4 Analysis of amastigotes inside BMMOs

Macrophages infected with trypomastigotes as described above (Treatment 1) were maintained in culture for four days, during, which time the parasites differentiated into amastigote forms. The infected macrophages were fixed with methanol, stained with Giemsa and analysed using Leica DMI 4000B microscope. The results are expressed as an average number of amastigotes per macrophage.

## Supplementary data

The following is the supplementary data (These data include additional details of HRMS, TLC analyses, experimental details of the alternative route to **7**, and ^1^H and ^13^C NMR spectra for cycloligomerisation products) related to this article:
