# Supplementary material for: Click chemistry oligomerisation of azido-alkyne-functionalised galactose accesses triazole-linked linear oligomers and macrocycles that inhibit Trypanosoma cruzi macrophage invasion
Source: Tetrahedron. 2015 Sep 30;71(39):7344–53. doi: 10.1016/j.tet.2015.04.085 (PMC4542550; doi:10.1016/j.tet.2015.04.085)
Supplement: Supplementary file 1 [file mmc1.pdf]

Supplementary Information

Click chemistry oligomerisation of azido-alkyne-functionalised galactose accesses triazole-linked linear oligomers and macrocycles that inhibit *Trypanosoma cruzi* macrophage invasion

Vanessa L. Campo,[a,±] Irina M. Ivanova,[b, ±] Ivone Carvalho,[a] Carla D. Lopes,[c] Zumira A. Carneiro,[c] Gerhard Saalbach,[b] Sergio Schenkman,[d] João Santana da Silva,[c] Sergey A. Nepogodiev[b] and Robert A. Field[b,*]

[a] *Faculdade de Ciências Farmacêuticas de Ribeirão Preto, USP, Av. Café S/N, CEP 14040-903, Ribeirão Preto, SP, Brazil*
[b] *Department of Biological Chemistry, John Innes Centre, Norwich Research Park, Norwich NR4 7UH, UK*
[c] *Faculdade de Medicina de Ribeirão Preto, Department of Parasitology Microbiology and Immunology, USP, Av. Bandeirantes 3900, CEP 14049-900, Ribeirão Preto, SP, Brazil*
[d] *Department of Microbiology, Immunology and Parasitology, Universidade Federal de São Paulo, Rua Botucatu 862 8, Andar 04023-062 São Paulo, SP, Brazil*

List of Tables, Schemes and Figures

**Table S1**	Yields of 1,4-triazole-linked cyclic and linear products from CuAAC reactions of monomer **7**	2
**Table S2**	HRMS data for products of enzymatic sialylation of triazole-linked cyclic galactooligomers	4
**Scheme S1.**	Alternative route to compound **7** based on BF_3•OEt_2-catalysed glycosylation with 1-acetate **24**.	4
**Fig. S1**	The HRMS spectrum of the series of 1,4-triazole-linked linear products	3
**Fig. S2**	The TLC analysis of enzymatic transformations of 1,4-triazole-linked cyclic monomer **8**, 1,5-triazole-linked cyclic monomer **19**, 1,4-triazole-linked cyclic dimer **9** and 1,4 triazole-linked cyclic trimer **10**	4
**Fig. S3**	1H NMR and 13C NMR spectra of compound **4**	6
**Fig. S4**	1H NMR and 13C NMR spectra of compound **5**	7
**Fig. S5**	1H NMR and 13C NMR spectra of compound **6**	8
**Fig. S6**	1H NMR and 13C NMR spectra of compound **7**	9
**Fig. S7**	1H NMR and 13C NMR spectra of compound **8**	10
**Fig. S8**	1H NMR and 13C NMR spectra of compound **9**	11
**Fig. S9**	1H NMR and 13C NMR spectra of compound **10**	12
**Fig. S10**	1H NMR and 13C NMR spectra of compound **11**	13
**Fig. S11**	1H NMR and 13C NMR spectra of compound **12**	14
**Fig. S12**	1H NMR and 13C NMR spectra of compound **13**	15
**Fig. S13**	1H NMR and 13C NMR spectra of compound **14**	16
**Fig. S14**	1H NMR and 13C NMR spectra of compound **19**	17
**Fig. S15**	1H NMR and 13C NMR spectra of compound **20**	18
**Fig. S16**	1H NMR and 13C NMR spectra of compound **26**	19

Table S1 Yields of 1,4-triazole-linked cyclic and linear products from CuAAC reactions of monomer **7**

Reaction products	Method A	Method B
	Yield (%)	Yield (%)
**8**	9.0	6.7
**9**	3.0	2.2
**10**	1.6	1.0
**11**	1.1	0.8
**12**	1.3	0.6
**13**		0.5
Cyclic high mol. weight compounds	13.7	6.0
Mixture of linear compounds	25.8	35.7

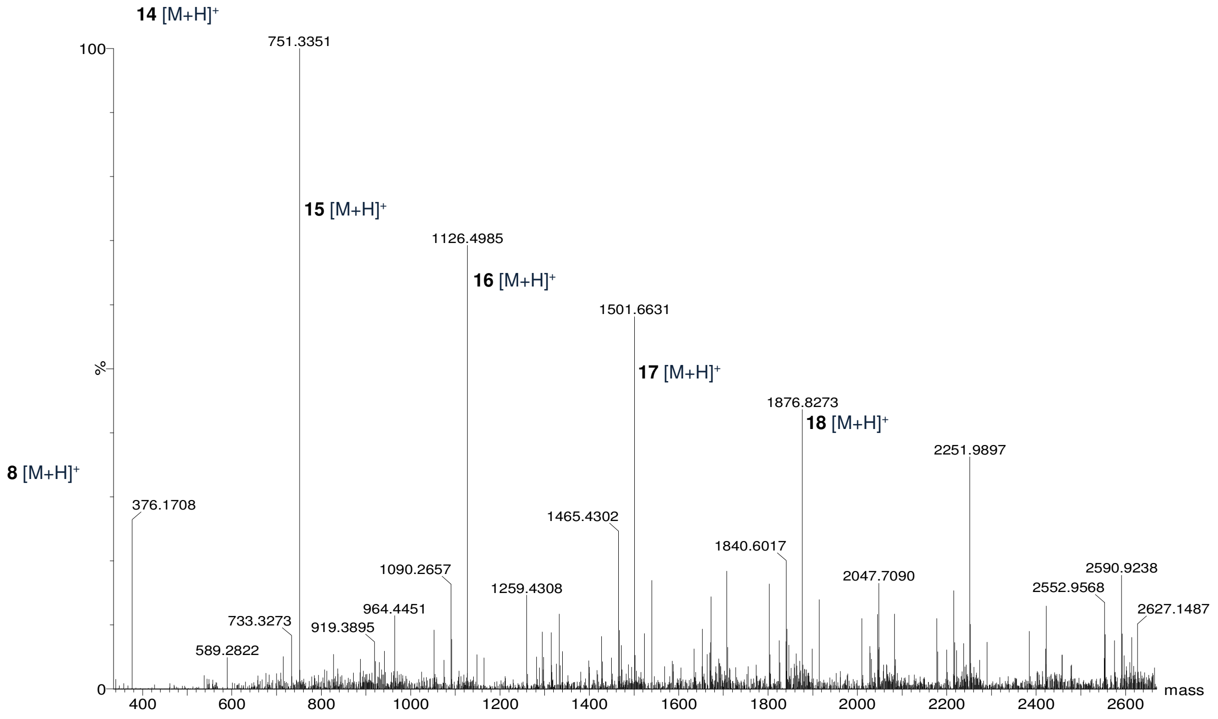

Fig. S1 The HRMS spectrum of the series of 1,4-triazole-linked linear products obtained from CuAAC oligomerisation of monomer **7**.

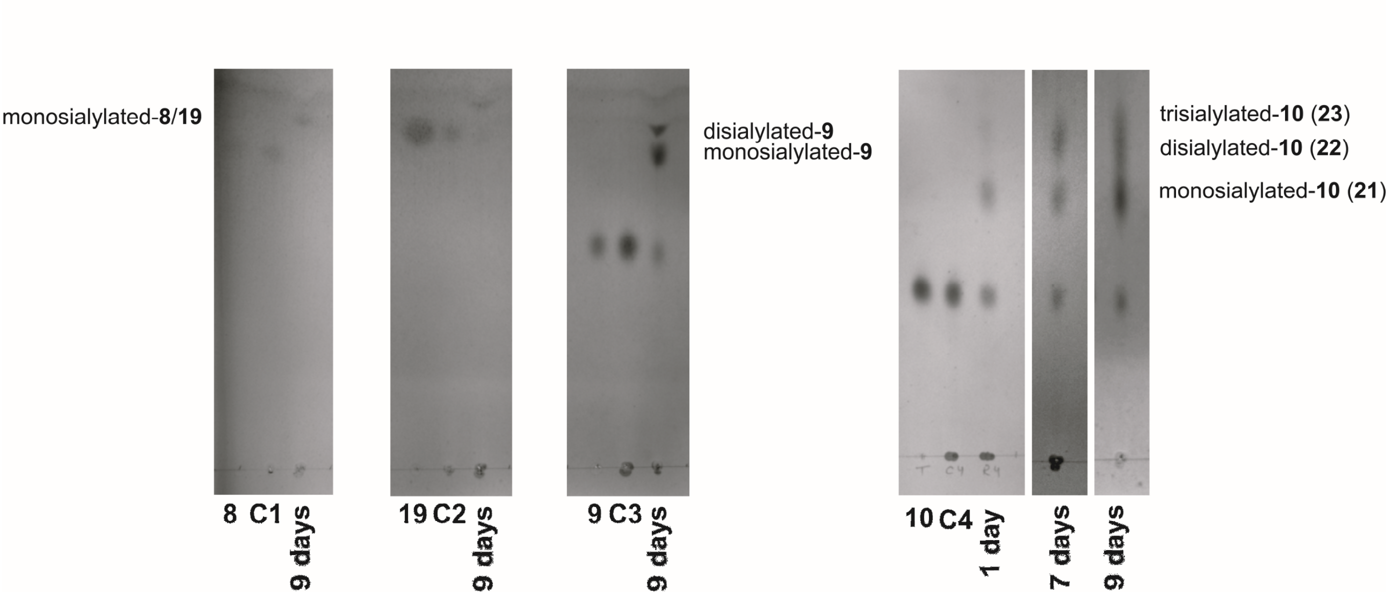

Fig. S2 The TLC analysis of enzymatic transformations of 1,4-triazole-linked cyclic monomer **8**, 1,5-triazole-linked cyclic monomer **19**, 1,4-triazole-linked cyclic dimer **9** and 1,4 triazole-linked cyclic trimer **10** into mono-, di- and tri-sialylated cyclic compounds in the presence of TcTS and a fetuin donor. Sialylated products were identified on TLC on the basis of the time course of enzymatic reactions. Negative controls (lanes **C1, C2, C3** and **C4**) were carried out in parallel with a donor and an acceptor without the enzyme. The progress of reactions was monitored by analysing samples after 1 day, 7 days and 9 days. TLC was eluted with CH_3CN/EtOAc/iPrOH/H_2O (85/25/50/50).

Table S2 HRMS data for products of enzymatic sialylation of triazole-linked cyclic galactooligomers

Starting	Mono-sialylated		Di-sialylated		Tri-sialylated	
material	Calcd *m/z*	Found *m/z*	Calcd *m/z*	Found *m/z*	Calcd *m/z*	Found *m/z*
Monomer **8**	665.2523 ([M-H]$^-$)	665.2519 ([M-H]$^-$)				
Monomer **19**	665.2523 ([M-H]$^-$)	665.2519 ([M-H]$^-$)				
Dimer **9**	1040.4164 ([M-H]$^-$)	1040.4237 ([M-H]$^-$)	1331.5118 ([M-H]$^-$)	13315272 ([M-H]$^-$)		
Trimer **10**	1415.5806 ([M-H]$^-$)	1415.5994 ([M-H]$^-$) (**21**)	1706.6760 ([M-H]$^-$)	1706.7021 ([M-H]$^-$) (**22**)	1997.7714 ([M-H]$^-$)	1997.7947([M-H]$^-$) (**23**)

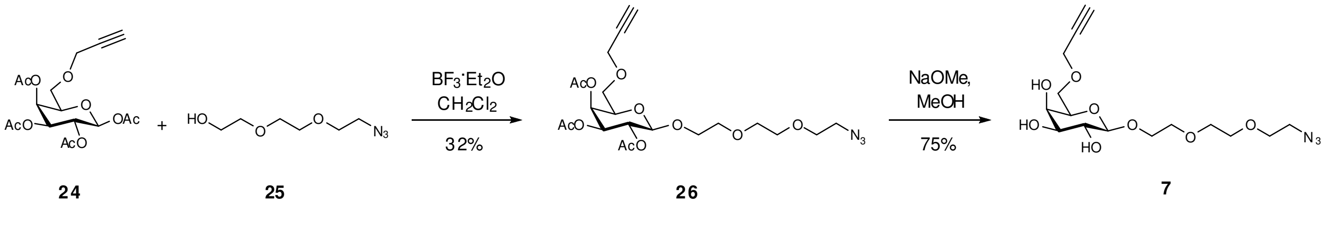

Scheme S1. Alternative route to compound **7** based on BF3•OEt2-catalysed glycosylation with 1-acetate **24**.

1. Experimental Section

1.1. 2-(2-(2-Azidoethoxy)ethoxy)ethanol (25)

A mixture of 2-(2-(2-chloroethoxy)ethoxy)ethanol (1g, 5.9 mmol) and NaN_3 (5.75 g, 88.5 mmol) in DMF (20 mL) was stirred for 9 h at room temperature. The solvent was removed under reduced pressure, the residue was resuspended in CH_2Cl_2 and filtered through Celite. The product **25** was obtained as an yellow oil, yield 1.03 g, (100%): FTIR (KBr) ν_{max}: 3417; 2869; 2104; 1666; 1066 cm^{-1}; δ_H ($CDCl_3$, 400 MHz) 3.76-3.60 (10H, m, CH_2O), 3.41 (2 H, t, *J* 5.0 Hz, $CH_2 N_3$).

1.2. 2-(2-(2-Azidoethoxy)ethoxy)ethyl) 2,3,4-tetra-*O*-acetyl-6-*O*-(prop-2-ynyl)-β-D-galactopyranoside (26)

To a solution of acetate **24** (200 mg, 0.52 mmol) and alcohol **25** (108 mg, 0.62 mmol) in CH_2Cl_2 (10 mL) BF_3•Et_2O (0.095 mL, 0.77 mmol) was added. The reaction mixture was stirred for 20 h at room temperature and then 6 h at 52 ºC. The resulting solution was diluted with CH_2Cl_2, washed with satd $NaHCO_3$ solution and brine, dried ($MgSO_4$), concentrated and purified by flash chromatography (EtOAc/Hex 1:1) to give compound **26** (83 mg, 32%). $[\alpha]_D^{25}$ + 18.2 (c 1.0 $CHCl_3$);. δ_H ($CDCl_3$, 400 MHz) 5.42 (1H, d, $J_{3,4}$=3.4 Hz, H-4), 5.20 (1H, dd, $J_{1,2}$=8.0 Hz; $J_{2,3}$=10.5 Hz, H-2), 5.02 (1H, dd, $J_{3,4}$=3.4 Hz; $J_{2,3}$=10,5 Hz, H-3), 4.56 (1H, d, $J_{1,2}$=8.1 Hz, H-1), 4.13 (2H, dd, *J*=2.4 Hz, *J*=6.1 Hz, C*H*2C≡CH), 3.97 (1H, m, H-5), 3.85 (1H, dd, $J_{5,6a}$=6.3 Hz, $J_{6,6b}$=12.4 Hz, H-6a), 3.76 (1H, dd, $J_{5,6b}$=6,3 Hz, $J_{6a,6b}$=12.2 Hz, H-6b), 3.70-3.60 (10H, m, CH_2), 3.4 (2H, t, *J*=5.2 Hz, CH_2N_3), 2.42 (1H, s, CH_2C≡C*H*), 2.4–1.9 (9H, 3s, Ac); δ_C ($CDCl_3$, 100 MHz) 170.2, 170.1, 169.5 0 (*C*OCH_3); 101.4 (C-1); 79.1 (CH_2*C*≡CH), 74.9 (CH_2C≡*C*H); 72.0 (C-5); 71.0 (C-3); 70.75, 70.7, 70.4, 70.0 (OCH_2), 69.1 (C-2), 69.0 (C-6), 67.7 (CH_2), 67.5 (C-4), 58.6 (*C*H2C≡CH), 50.7 (CH_2N_3), 20.8, 20.7, , 20.6 (CO*C*H_3); HRMS (ESI): *m/z* calcd for $C_{21}H_{31}N_3O_{11}Na$ ($[M+Na]^+$): 524.1851, found: 524.1841

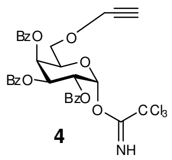

4

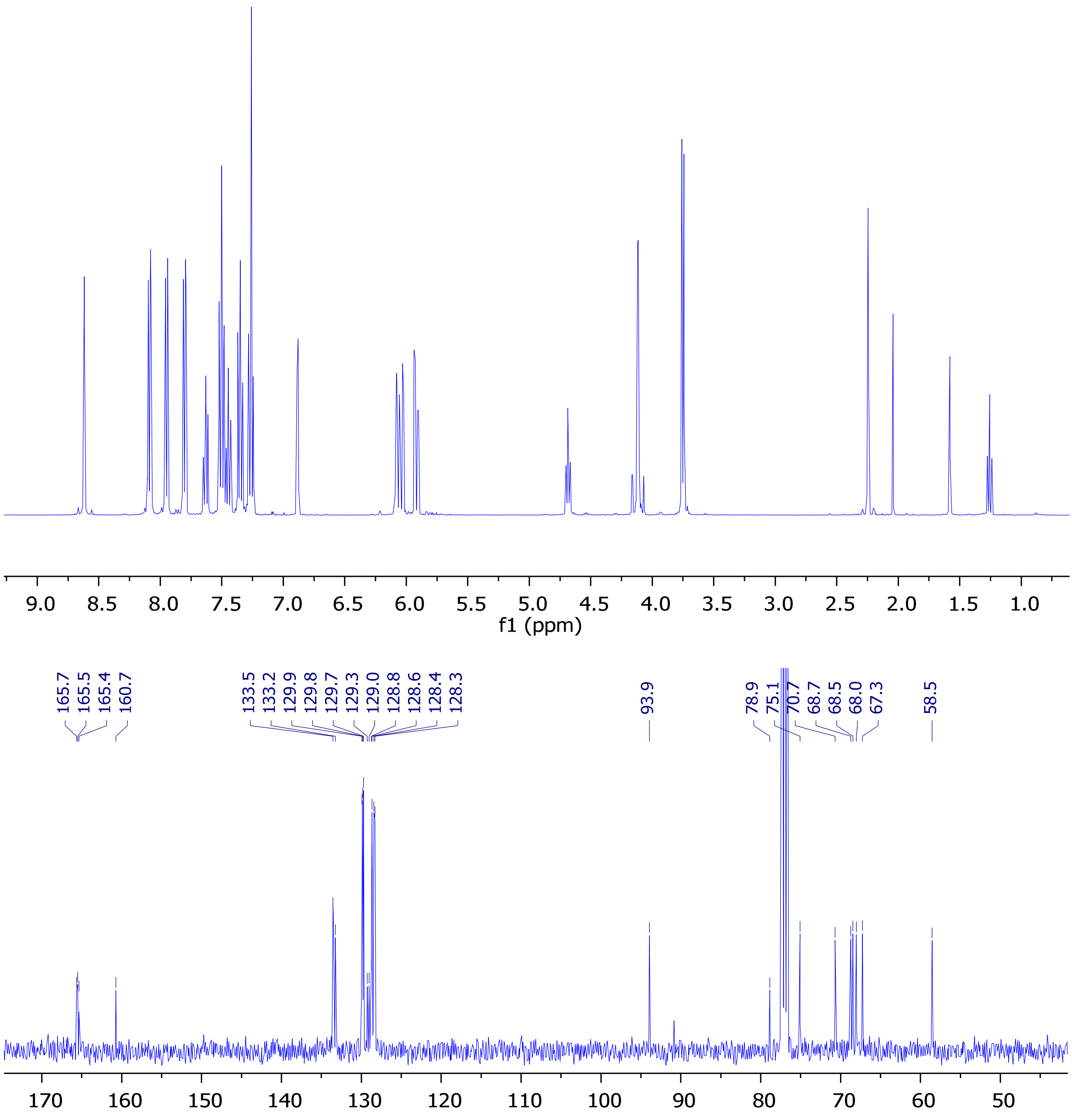

Fig. S3 ^{1}H NMR and ^{13}C NMR spectra of compound **4**.

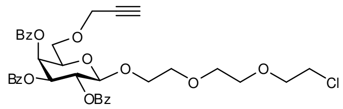

5

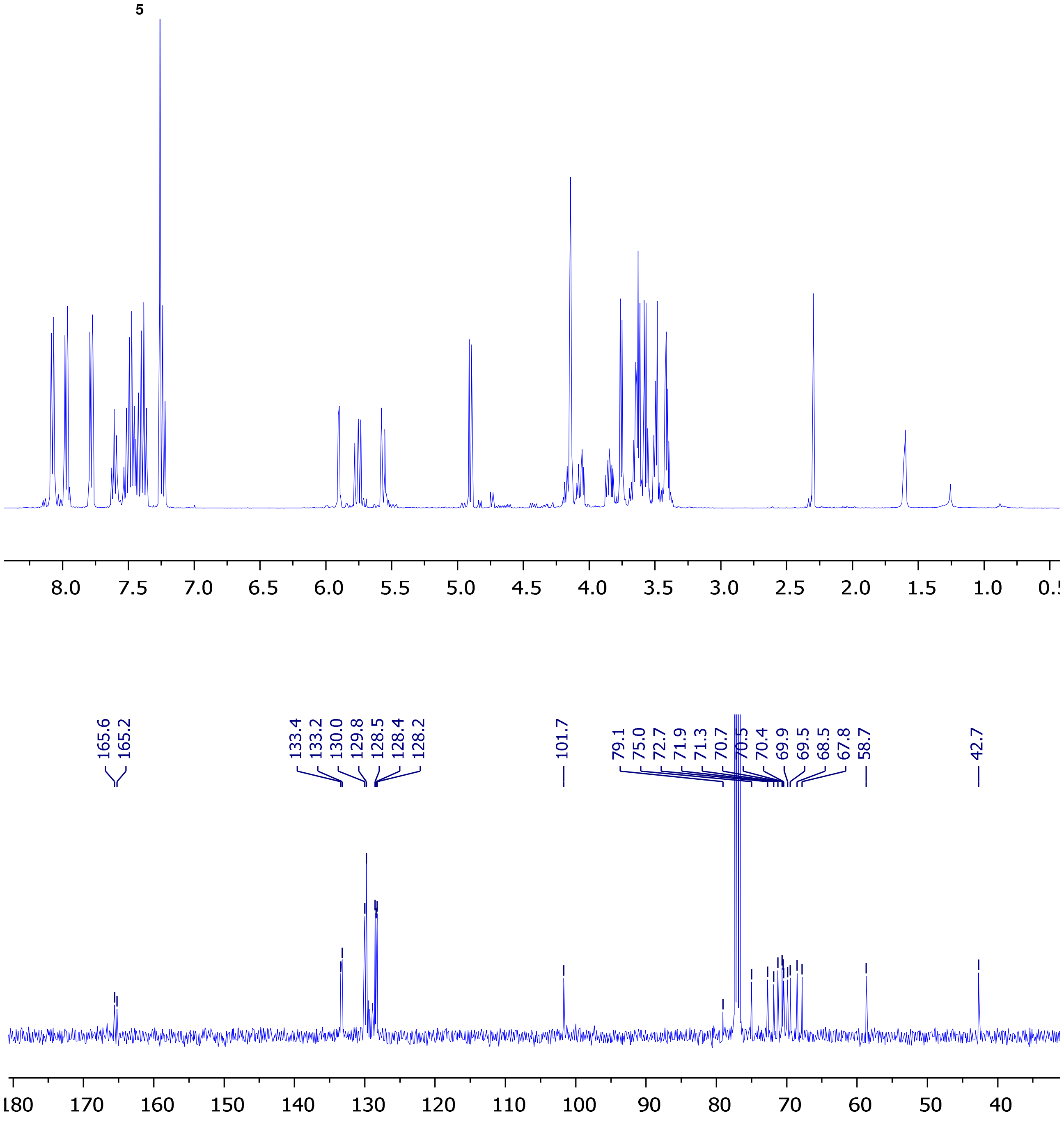

Fig. S4 ^{1}H NMR and ^{13}C NMR spectra of compound **5**.

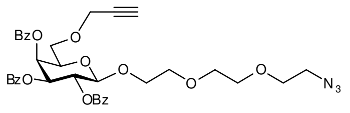

6

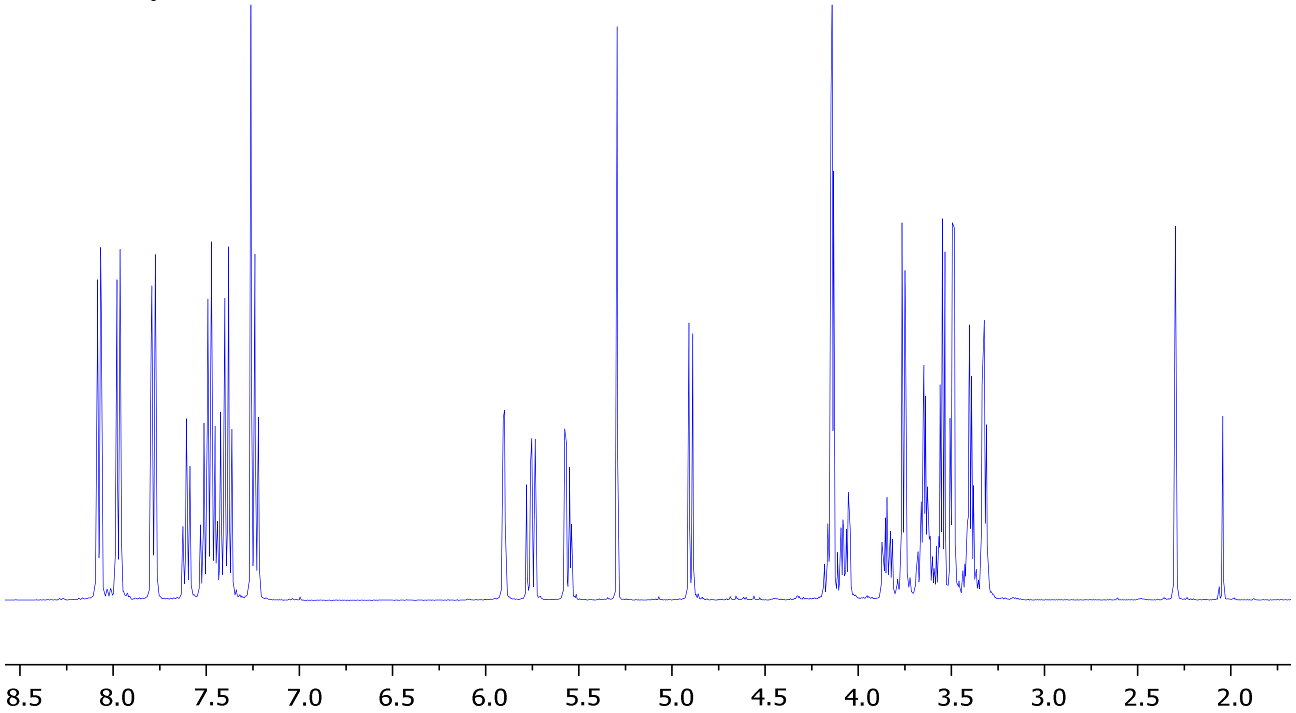

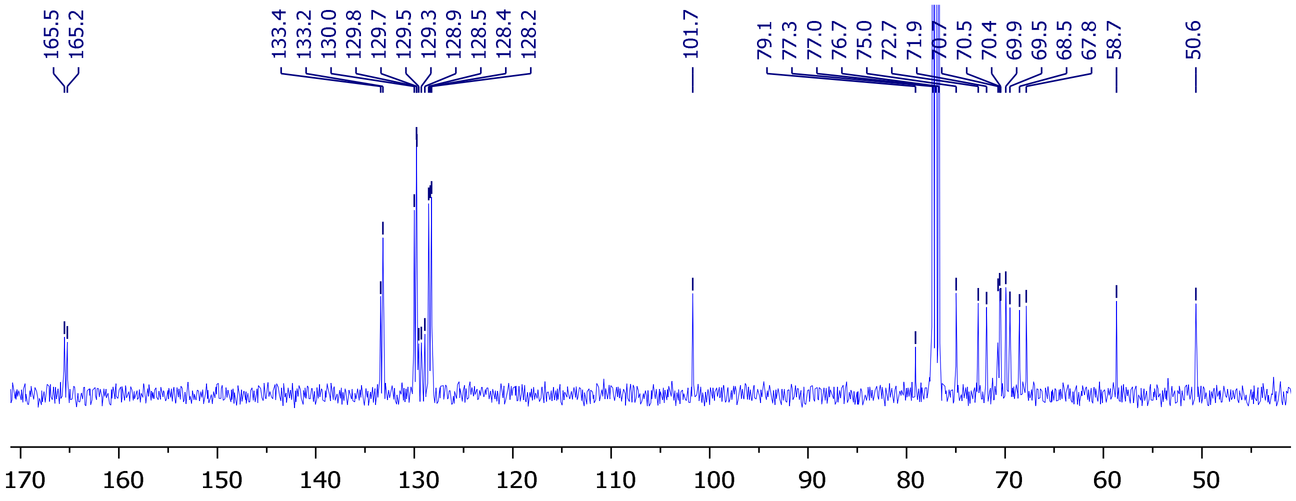

Fig. S5 ^{1}H NMR and ^{13}C NMR spectra of compound **6**.

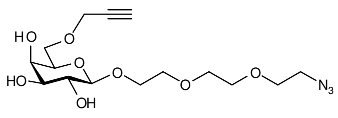

7

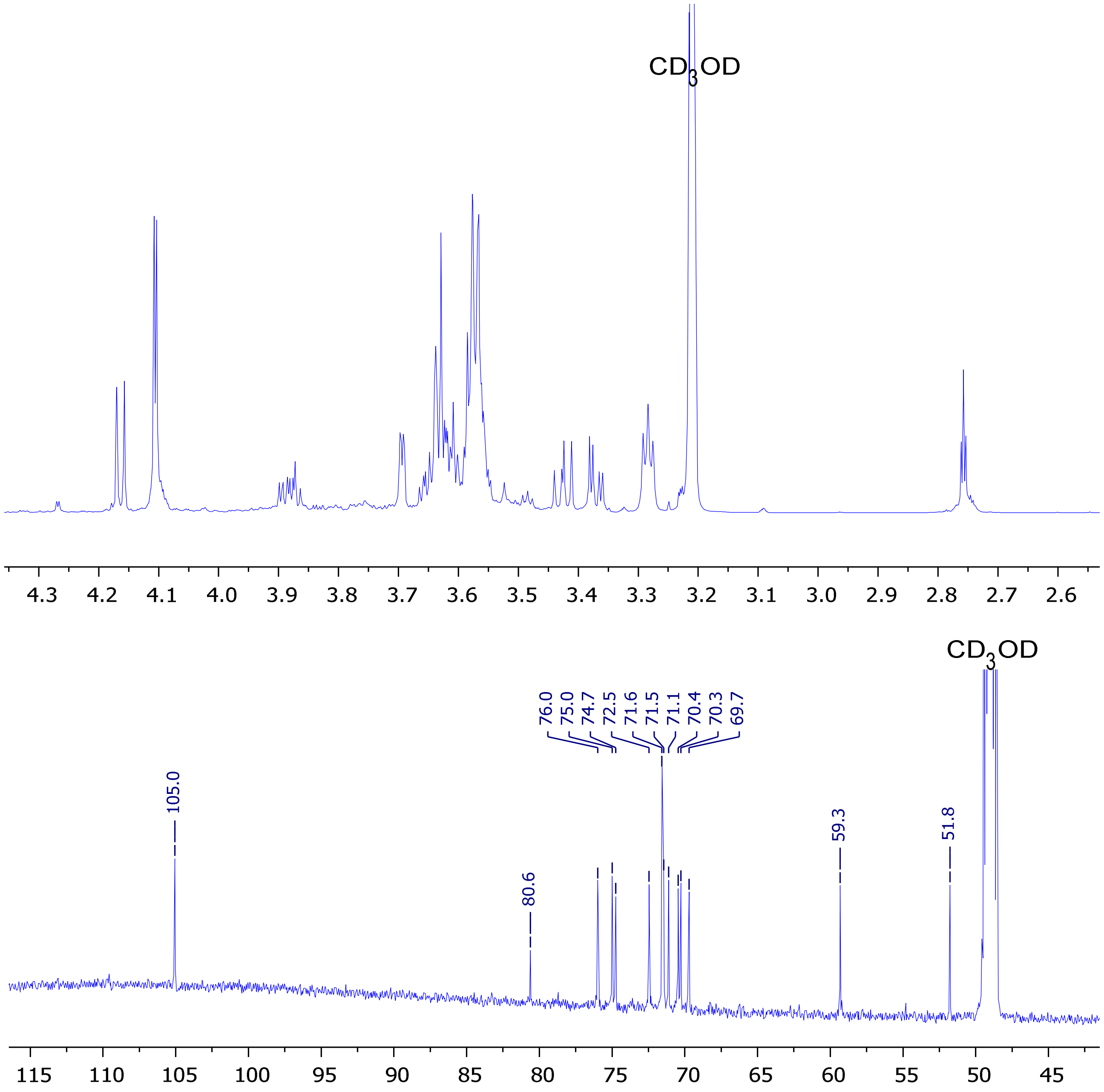

Fig. S6 ^{1}H NMR and ^{13}C NMR spectra of compound **7**.

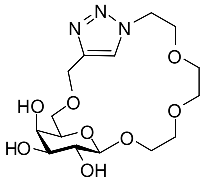

8

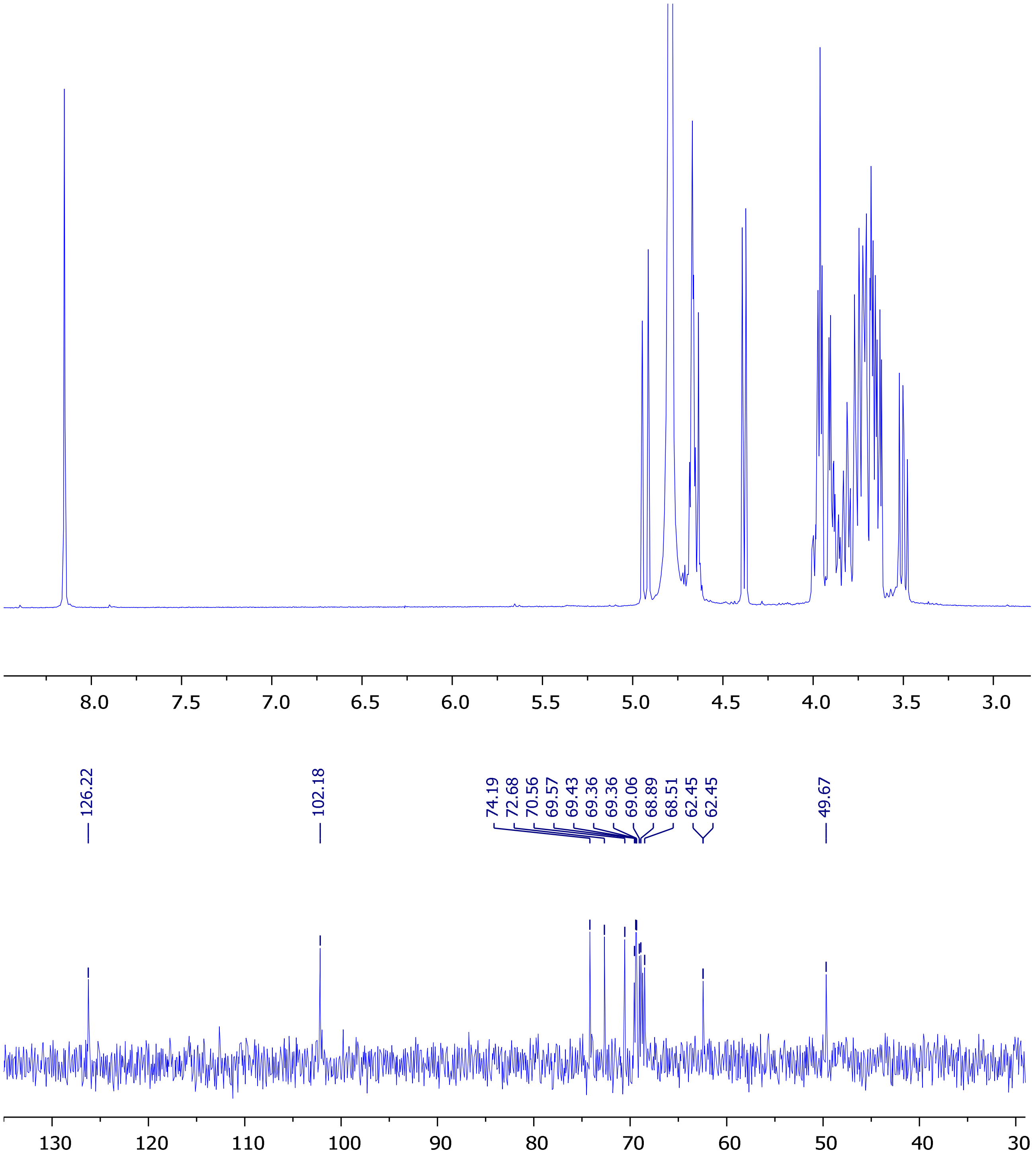

Fig. S7 ^{1}H NMR and ^{13}C NMR spectra of compound **8**.

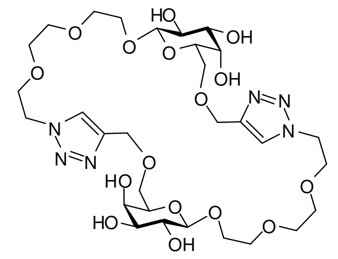

9

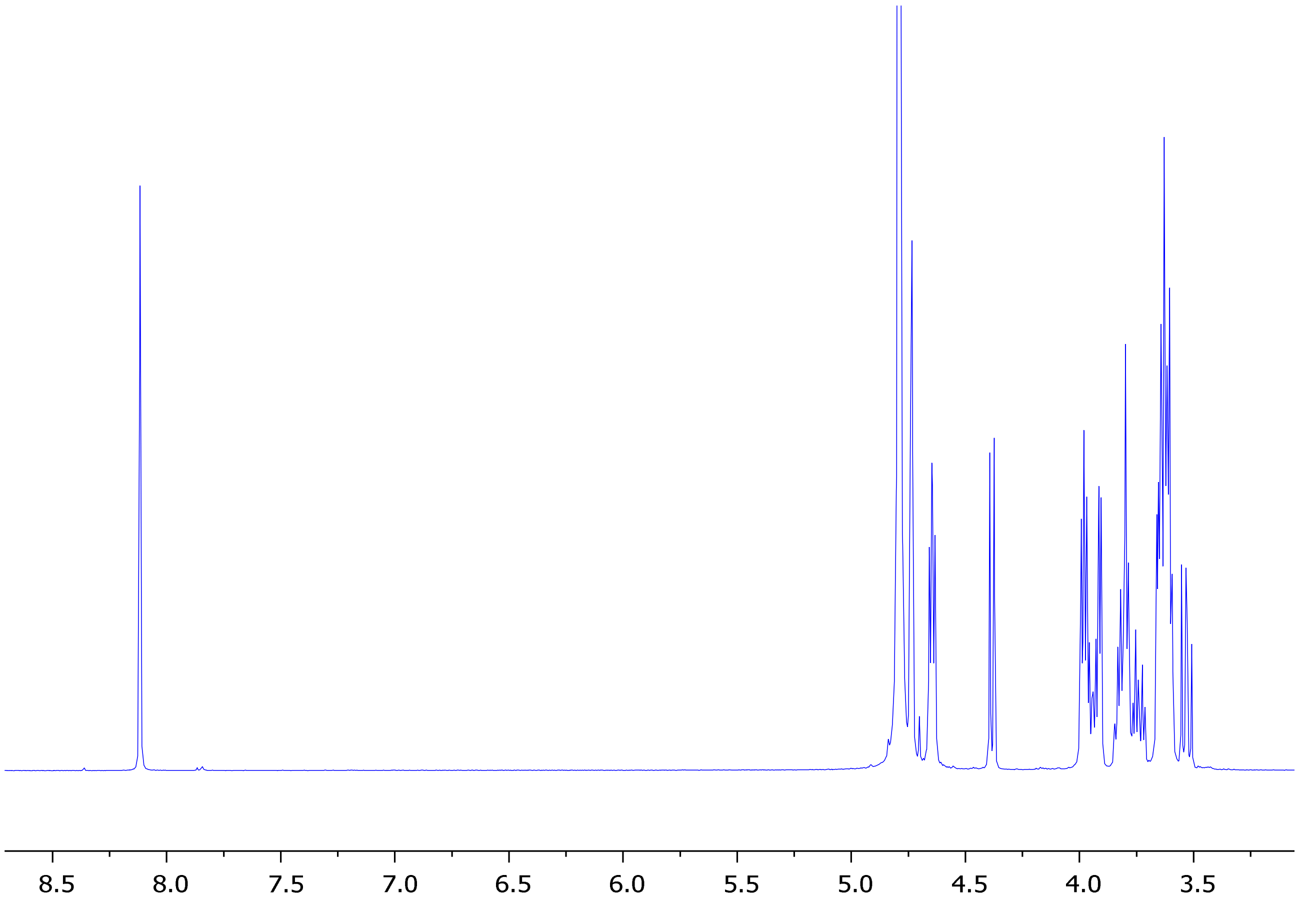

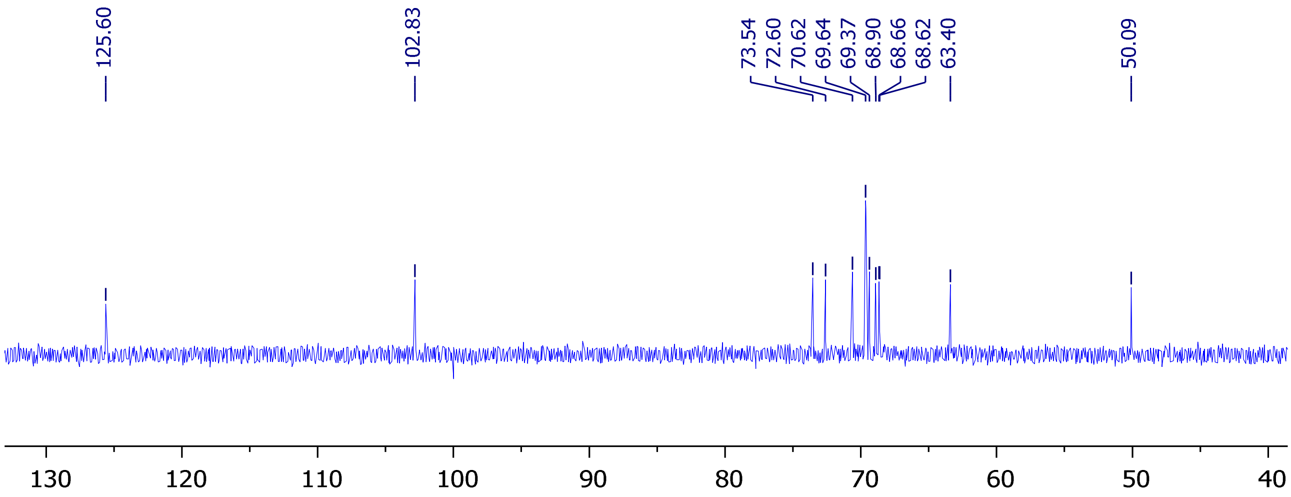

Fig. S8 ^{1}H NMR and ^{13}C NMR spectra of compound **9**b.

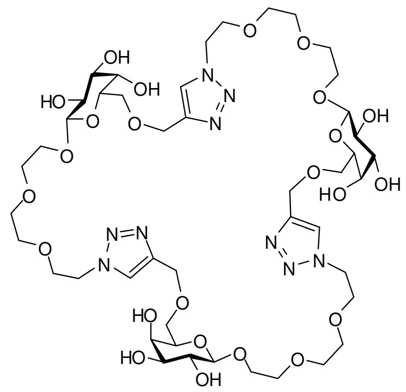

10

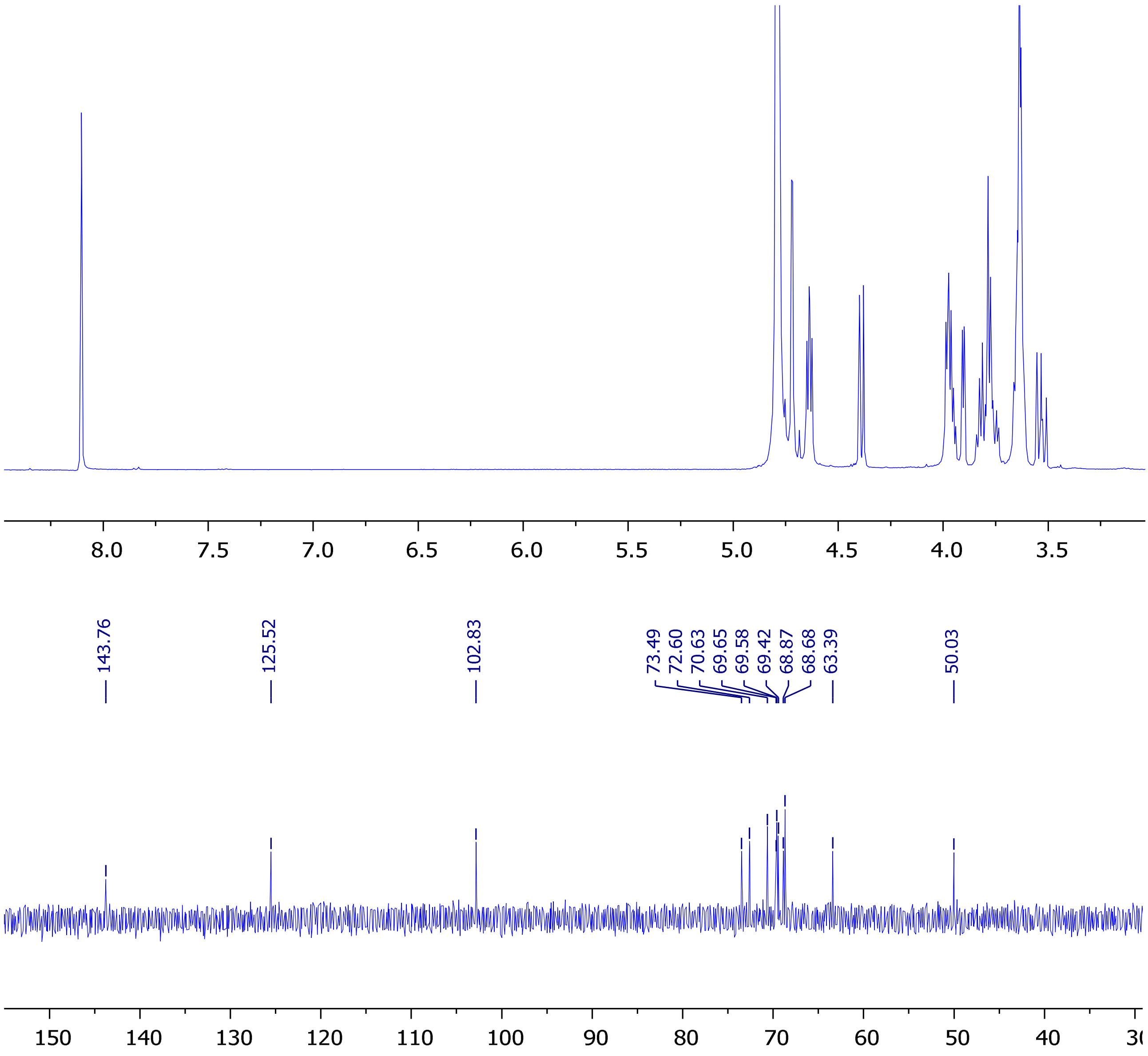

Fig. S9 ^{1}H NMR and ^{13}C NMR spectra of compound **10**.

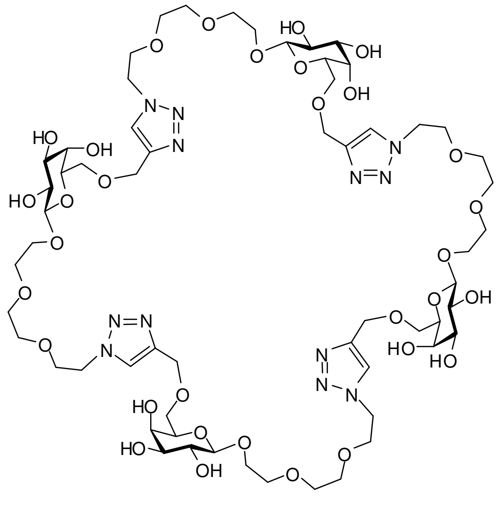

11

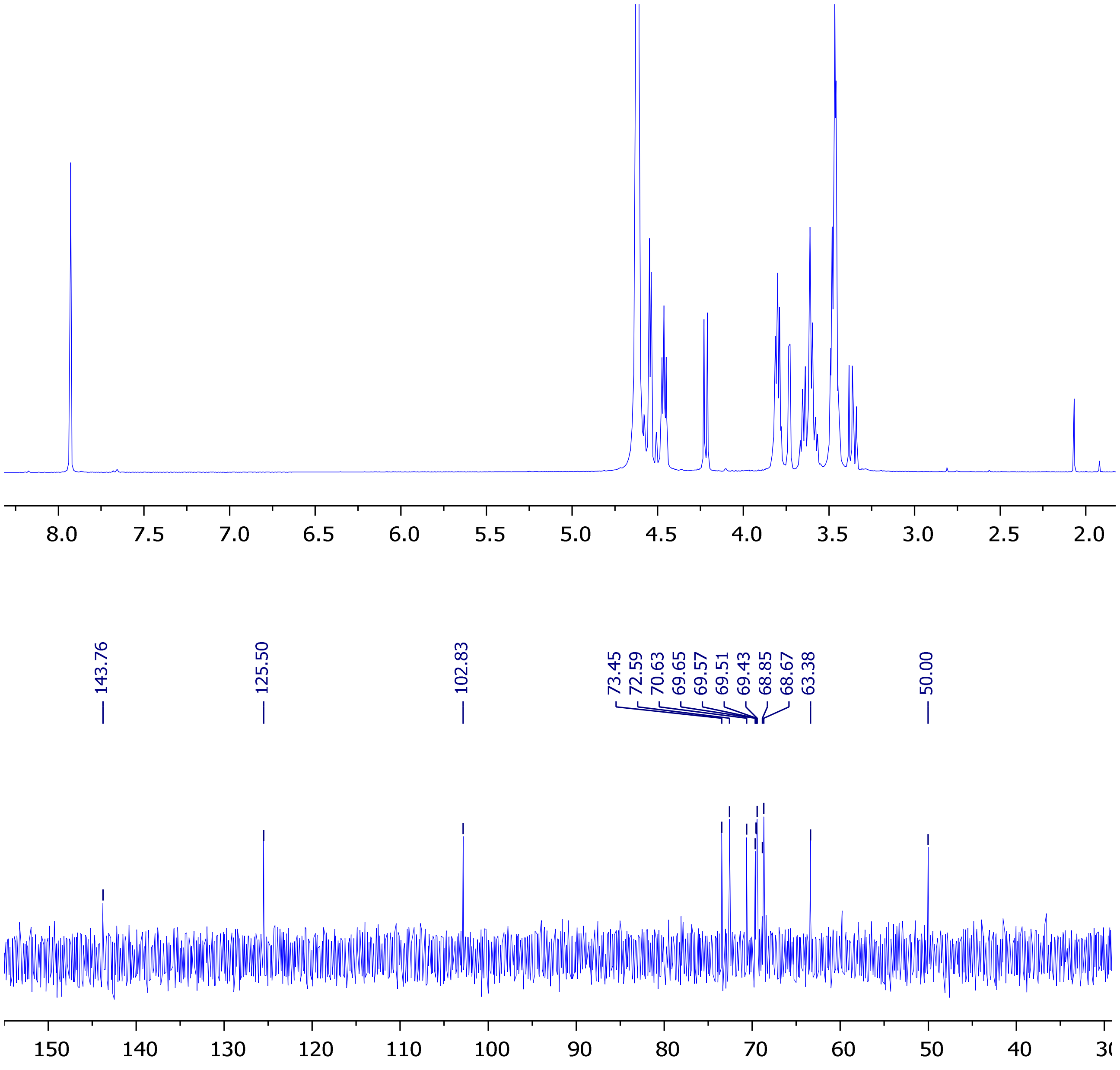

Fig. S10 ^{1}H NMR and ^{13}C NMR spectra of compound **11**.

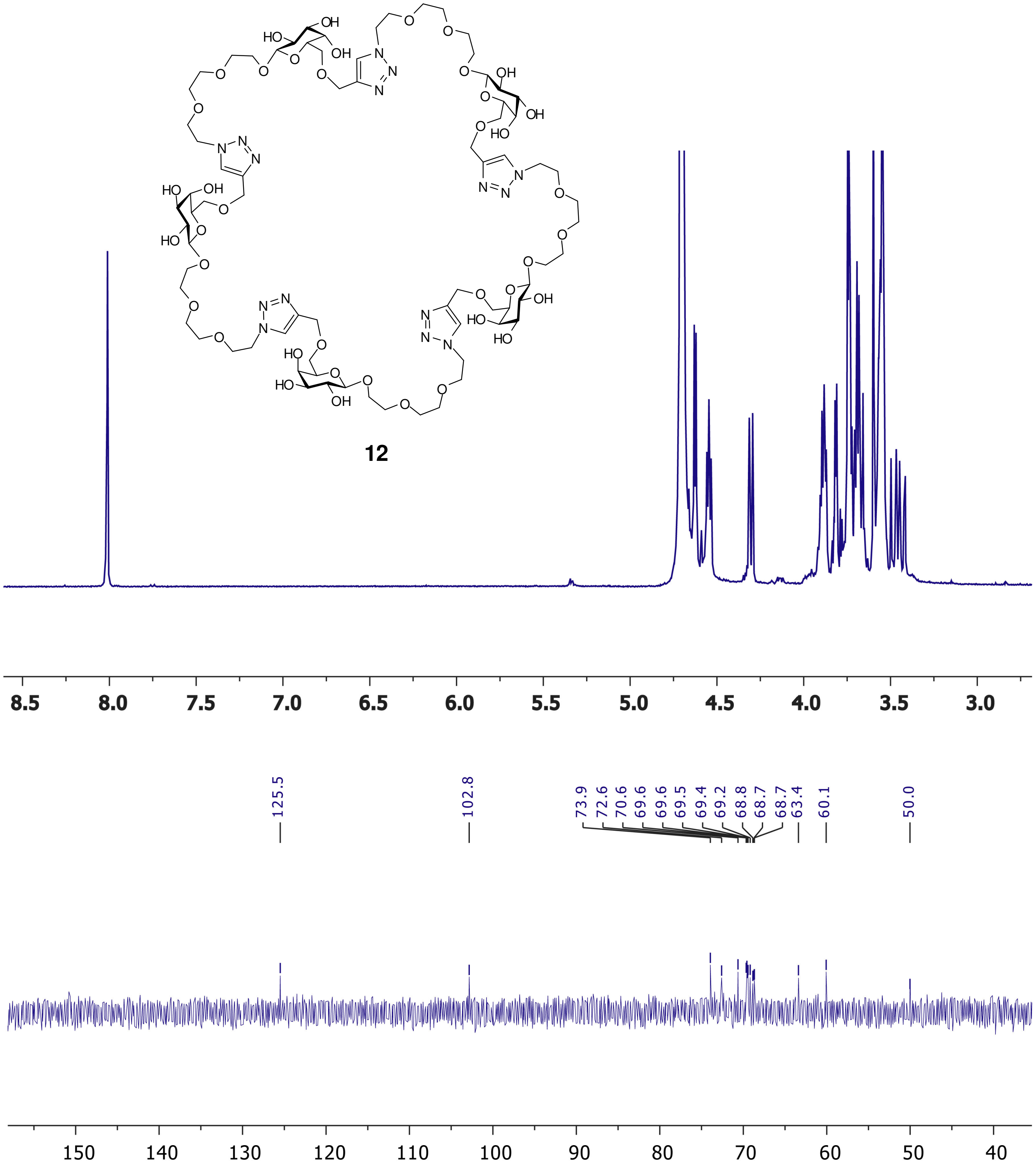

Fig. S11 ^{1}H NMR and ^{13}C NMR spectra of compound **12**.

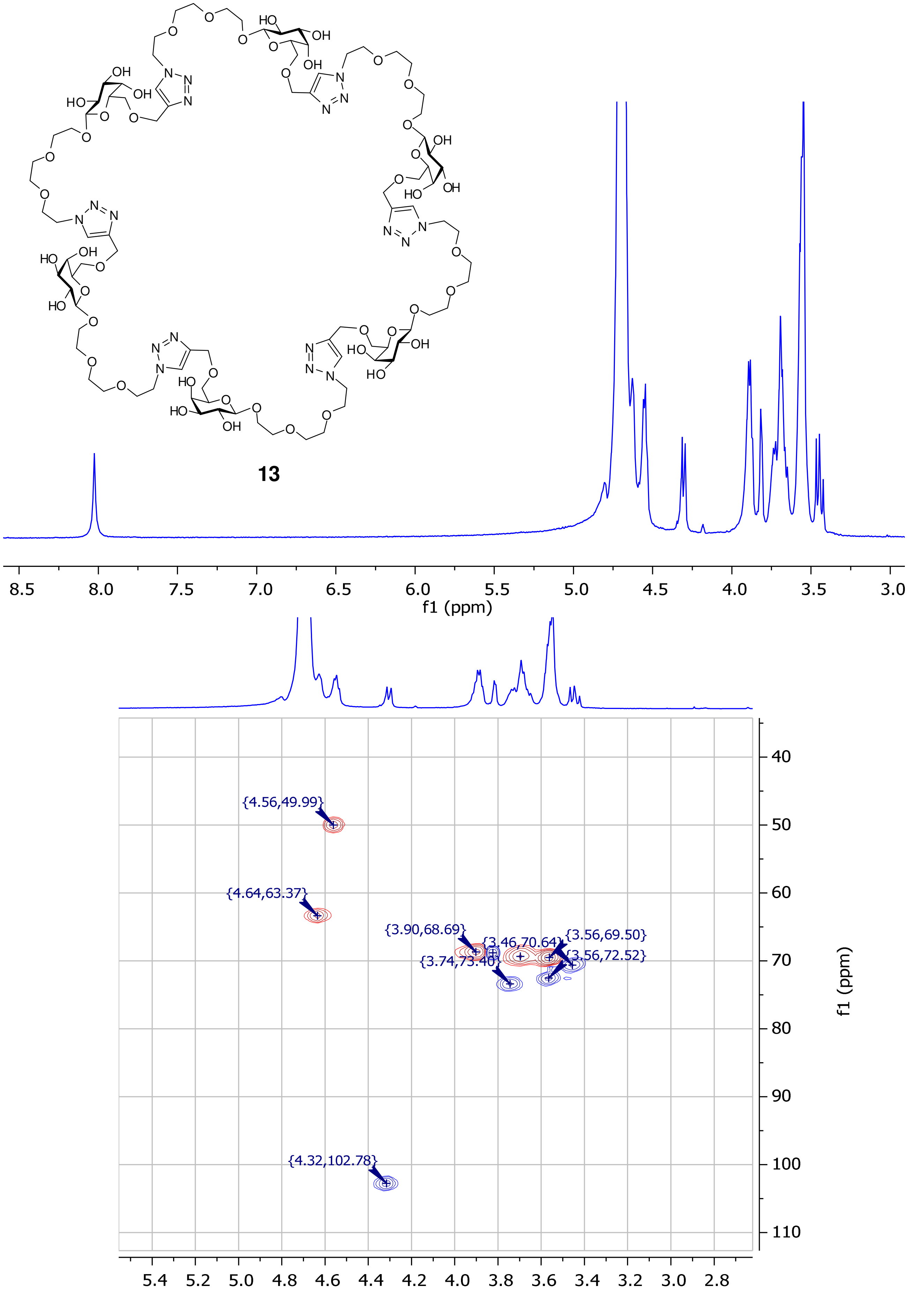

Fig. S12 ^{1}H NMR and ^{13}C NMR spectra of compound **13**.

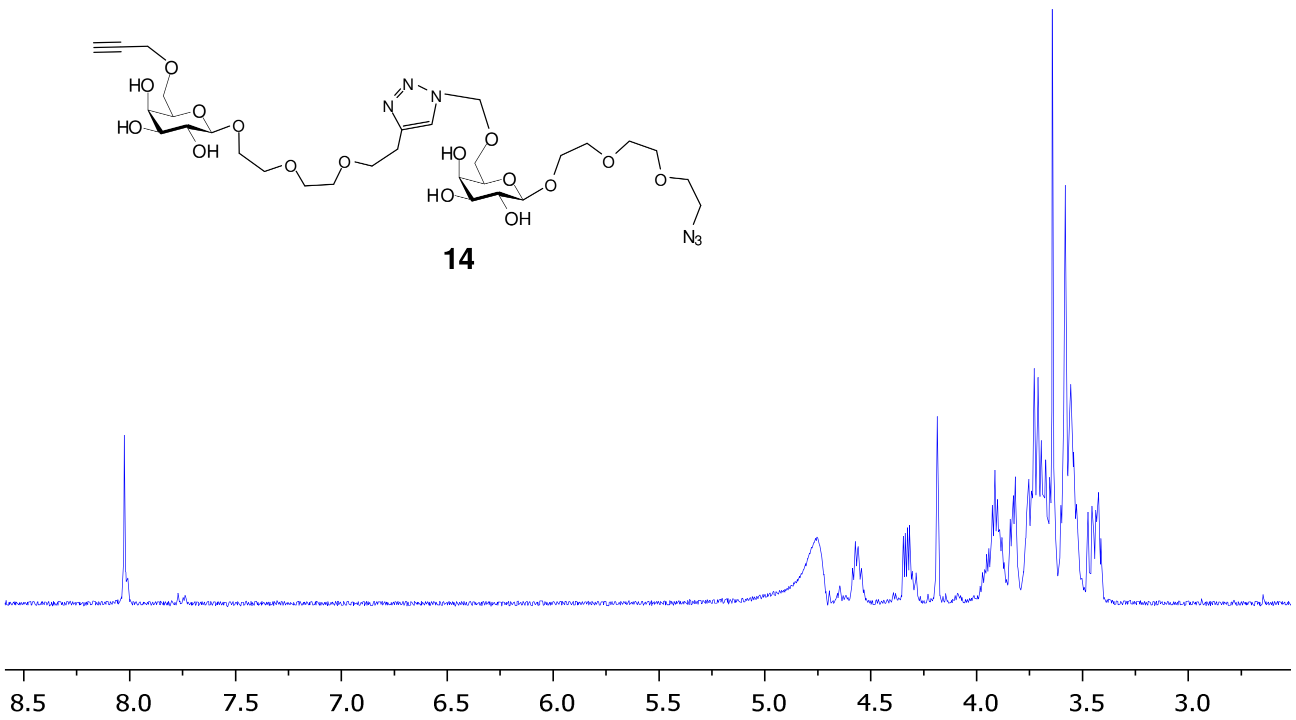

14

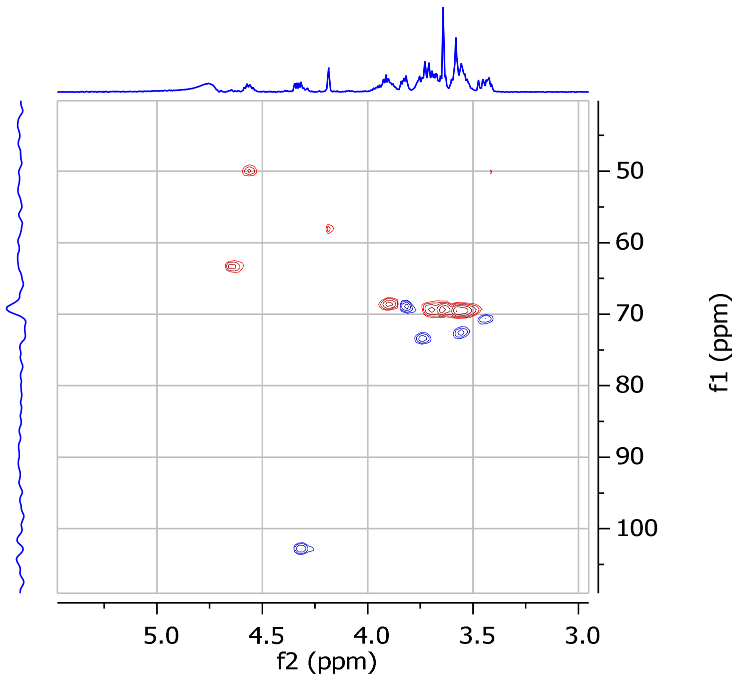

Fig. S13 ^{1}H NMR and ^{13}C NMR spectra of compound **14**.

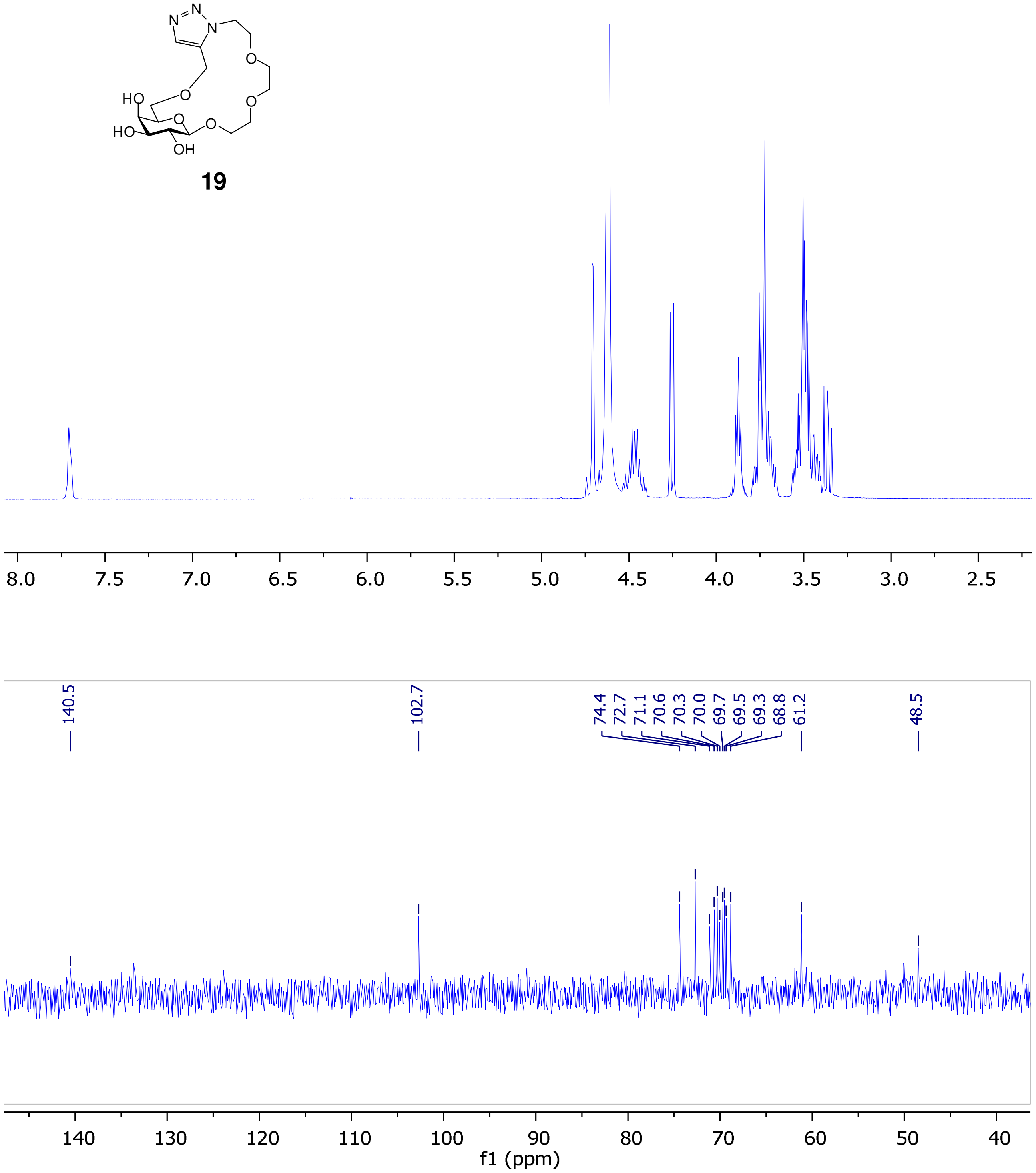

Fig. S14 ^{1}H NMR and ^{13}C NMR spectra of compound **19**.

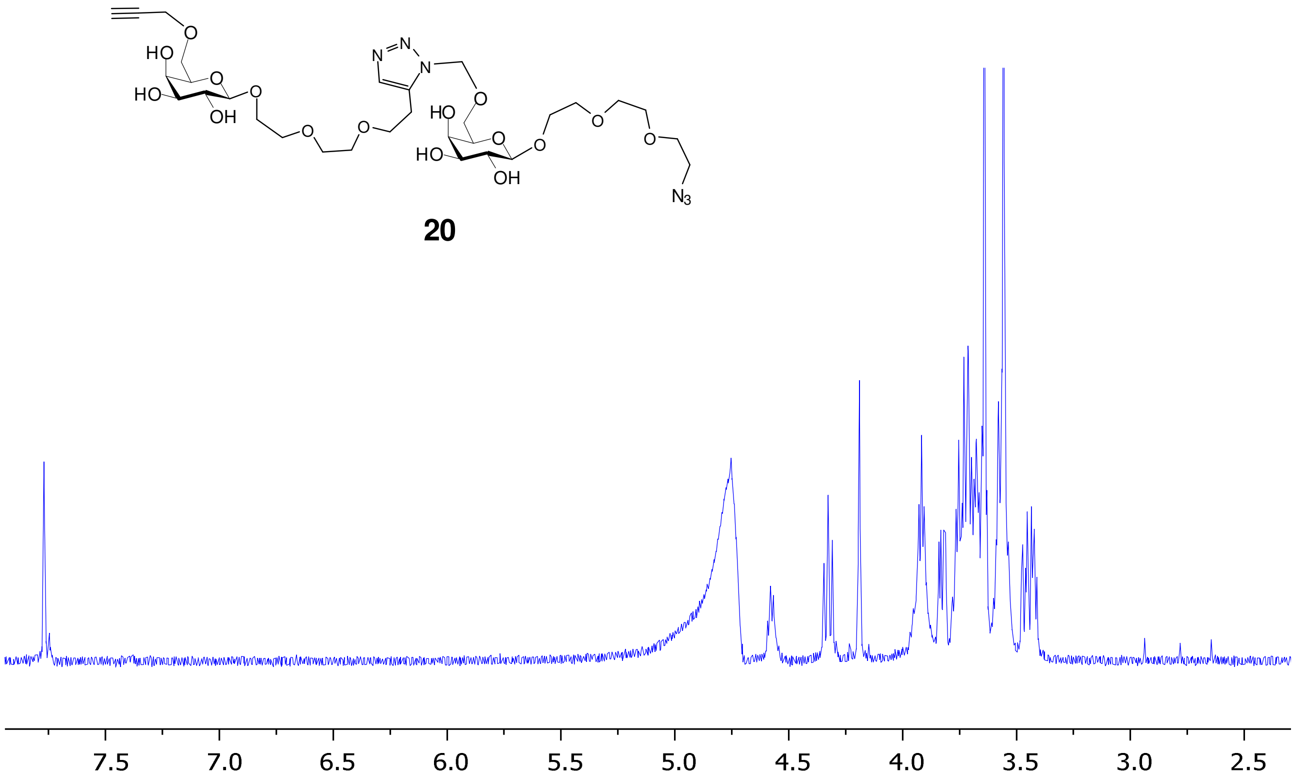

20

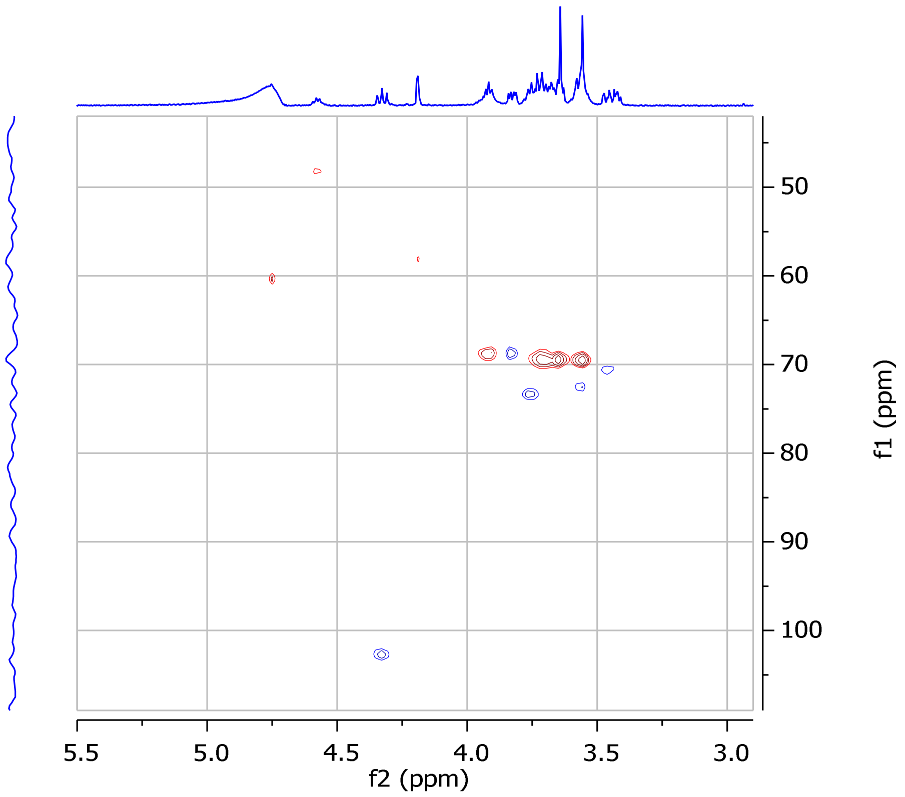

Fig. S15 ^{1}H NMR and ^{13}C NMR spectra of compound **20**.

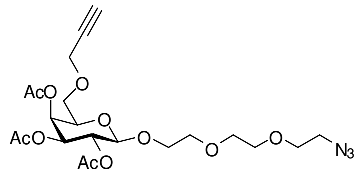

26

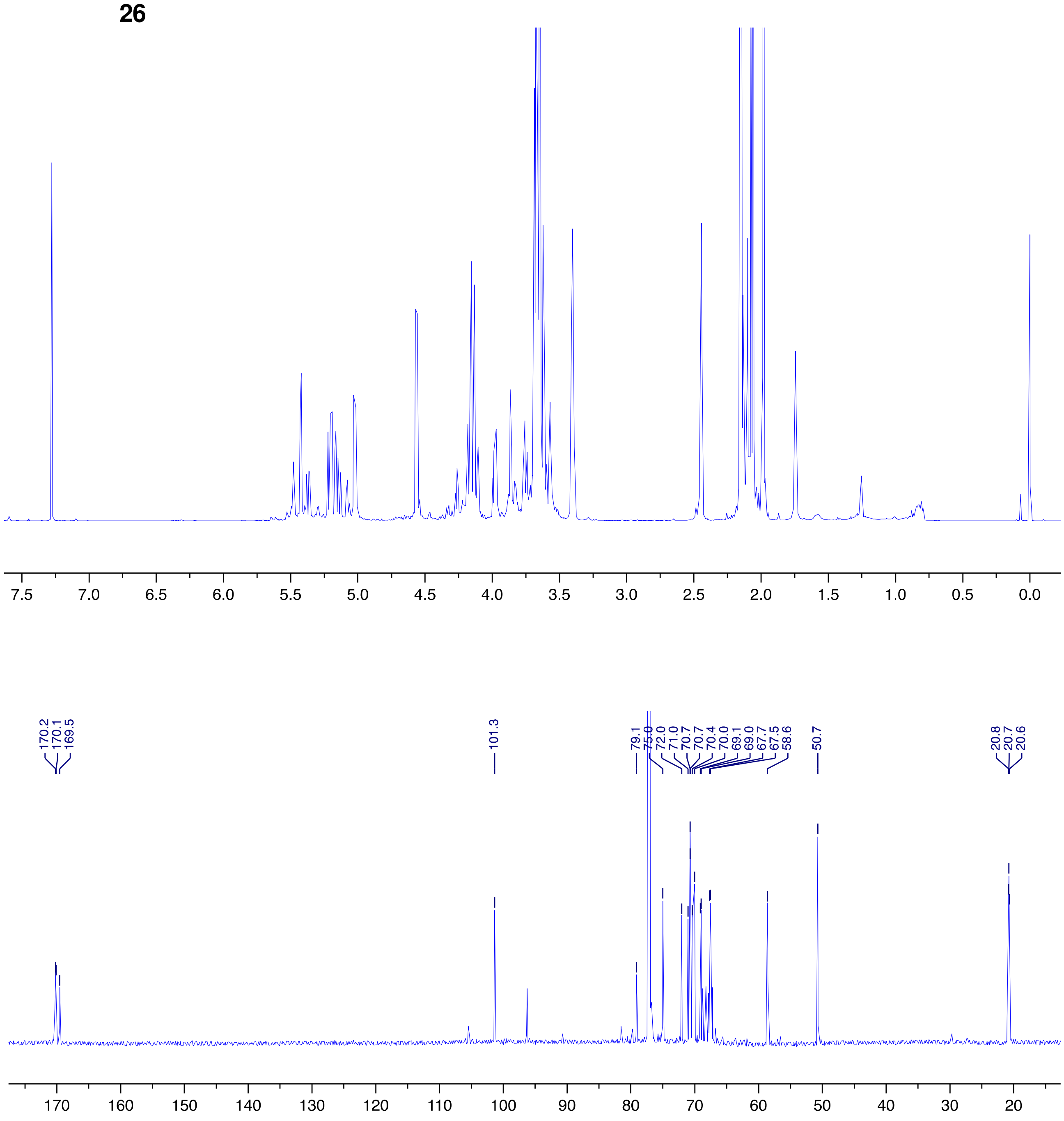

Fig. S16 ^{1}H NMR and ^{13}C NMR spectra of compound **13**.
